# Implications of Mitigating Ozone and Fine Particulate Matter Pollution in the Guangdong‐Hong Kong‐Macau Greater Bay Area of China Using a Regional‐To‐Local Coupling Model

**DOI:** 10.1029/2021GH000506

**Published:** 2022-03-11

**Authors:** Xuguo Zhang, Jenny Stocker, Kate Johnson, Yik Him Fung, Teng Yao, Christina Hood, David Carruthers, Jimmy C. H. Fung

**Affiliations:** ^1^ Department of Mathematics The Hong Kong University of Science and Technology Hong Kong China; ^2^ Division of Environment and Sustainability The Hong Kong University of Science and Technology Hong Kong China; ^3^ Cambridge Environmental Research Consultants Cambridge UK

**Keywords:** street‐scale, air dispersion model, CMAQ–ADMS‐urban, sensitivity analysis, ozone, Greater Bay Area

## Abstract

Ultrahigh‐resolution air quality models that resolve sharp gradients of pollutant concentrations benefit the assessment of human health impacts. Mitigating fine particulate matter (PM_2.5_) concentrations over the past decade has triggered ozone (O_3_) deterioration in China. Effective control of both pollutants remains poorly understood from an ultrahigh‐resolution perspective. We propose a regional‐to‐local model suitable for quantitatively mitigating pollution pathways at various resolutions. Sensitivity scenarios for controlling nitrogen oxide (NO_x_) and volatile organic compound (VOC) emissions are explored, focusing on traffic and industrial sectors. The results show that concurrent controls on both sectors lead to reductions of 17%, 5%, and 47% in NO_x_, PM_2.5_, and VOC emissions, respectively. The reduced traffic scenario leads to reduced NO_2_ and PM_2.5_ but increased O_3_ concentrations in urban areas. Guangzhou is located in a VOC‐limited O_3_ formation regime, and traffic is a key factor in controlling NO_x_ and O_3_. The reduced industrial VOC scenario leads to reduced O_3_ concentrations throughout the mitigation domain. The maximum decrease in median hourly NO_2_ is >11 μg/m³, and the maximum increase in the median daily maximum 8‐hr rolling O_3_ is >10 μg/m³ for the reduced traffic scenario. When controls on both sectors are applied, the O_3_ increase reduces to <7 μg/m³. The daily averaged PM_2.5_ decreases by <2 μg/m³ for the reduced traffic scenario and varies little for the reduced industrial VOC scenario. An O_3_ episode analysis of the dual‐control scenario leads to O_3_ decreases of up to 15 μg/m³ (8‐hr metric) and 25 μg/m³ (1‐hr metric) in rural areas.

## Key Points

- A regional‐to‐local coupled model is constructed to explore the likely impact of emissions reductions and pollution mitigation pathways
- The O_3_ formation regime in Guangzhou is VOC‐limited and the traffic sector is of paramount importance for controlling NO_x_ and O_3_
- Investigation of frequent summer O_3_ episodes emphasizes the value of more stringent VOC controls, particularly for the industrial sector

## 1 Introduction

Air pollution has attracted substantial research interests in recent years owing to its adverse effects on human health (Che et al., 2020; Conibear et al., 2021; Wu et al., 2019) and climate change (Li, Zhang, et al., 2019; Qin et al., 2017). Since the Chinese government announced a bold pledge that Chinese carbon emissions would peak before 2030 and China would achieve carbon neutrality by 2060, greater efforts have been made to alleviate air pollution (Cheng et al., 2021; Cui et al., 2021). The Chinese government has devoted tremendous efforts and released a series of emissions control policies to address this challenge (Cai et al., 2018; Jiang et al., 2015; Wu et al., 2019; Zhang et al., 2020). Zhang et al. (2020) proposed a holistic emissions control system that utilized a chemical transport model method to assess the impacts of the implemented emissions control policies in various sectors during the thirteenth Five‐Year‐Plan in China. In response to stringent national controls, ambient fine particulate matter (PM_2.5_) pollution has decreased substantially, whereas ozone (O_3_) pollution levels are becoming increasingly severe (Li, Jacob, Liao, Zhu, et al., 2019; Zhao et al., 2021). The combined impact of PM_2.5_ and O_3_ on human health has improved over the past decade (Zhang, Fung, Lau, Hossain, et al., 2021); however, high O_3_ concentrations have an adverse effect on ecosystems (Yli‐Pelkonen et al., 2017), so long‐term controls of atmospheric O_3_ are necessary. As such, strategies for effectively controlling the absolute concentrations of O_3_ and PM_2.5_ simultaneously in urban regions are of increasing interest and importance.

A growing number of studies have devoted great efforts to the mechanism of coupled O_3_ and PM_2.5_ pollution levels in regions throughout China. Li, Jacob, Liao, Shen, et al. (2019) found that the main cause of increasing O_3_ concentrations in the North China Plain (NCP) after 2013 was a significant reduction in PM_2.5_ concentrations, which slowed hydroperoxy radical consumption and increased the rate of O_3_ formation. Zhao et al. (2021) confirmed the interactions between the two pollutants and called for their concurrent control following an analysis of 4‐year observational data in China. Li, Jacob, Liao, Zhu, et al. (2019) proposed aggressive reductions of nitrogen oxide (NO_x_) and volatile organic compound (VOC) emissions to control air pollution in the NCP following analysis of summer O_3_ surface data collected during 2013–2018. Gong et al. (2021) utilized the Community Multiscale Air Quality (CMAQ) model at a 12‐km resolution to trace the precursors of PM_2.5_ and O_3_; exploring the regional effects of pollution transport on the interaction among the PM_2.5_ and O_3_ from cities in the Yangtze River Delta (YRD) region. In addition, using the same CMAQ model, Li, Hu, et al. (2021) concluded that industrial and traffic emissions were the dominant sources of both pollutants in the YRD. These findings have motivated further studies on the impact of precursors NO_x_ and VOC emissions from traffic and industrial sources in China.

Other studies have focused on quantifying the effects of model resolutions on air pollution simulations and health risks. A study conducted in the United States proved that a CMAQ model with finer resolution (4‐km vs. 12‐km grid spacing) was better for estimating the effects on health in urban areas; the results for rural areas were comparable for both 4‐ and 12‐km resolutions (Jiang & Yoo, 2018). Tao et al. (2020) found that finer‐resolution modeling could better capture and reproduce the temporal trends and magnitudes of meteorological conditions and air quality in Beijing. In contrast, a localized study indicated that grid resolution had little effect on PM_2.5_ and O_3_ simulations in the YRD (Wang et al., 2021). Liu et al. (2020) demonstrated that model resolution did not significantly improve predictions of PM_2.5_ and daily maximum 8‐hr O_3_ in Nanjing. However, the spatial distributions of both pollutants were better captured by a finer resolution model, leading to a >20% difference in estimates of premature mortality due to O_3_ exposure (Liu et al., 2020). The impact of model resolutions on pollutant simulations and estimates of health risks has varied across different cities and regions (urban or rural). Consequently, exploring whether higher‐resolution modeling techniques benefit model simulations for highly urbanized cities is of particular interest.

Previous studies have typically used a coarse‐resolution (>1 km) model and an observation‐oriented method to explore pollution sources or estimate health impacts for O_3_ and PM_2.5_ in the coupled systems (Li, Xu, et al., 2021; Silver et al., 2020). One study (Silveira et al., 2019) summarized a large number of coupled regional‐to‐local models that have been applied in urban regions worldwide; however, model calculation algorithms and assumptions have varied widely among other studies. The regional‐to‐local scale coupling system used in the current study follows the approach introduced in the studies by Hood et al. (2018) and Stocker et al. (2012). The concentrations of the coupled CMAQ–Urban Atmospheric Dispersion Modeling System (CMAQ–ADMS‐Urban) are calculated by an equation: *C*_coupled_ = *C*_CMAQ_ − *C*_ADMS‐U‐Grid_ + *C*_ADMS‐U‐Explicit_, where *C*_CMAQ_ represents the regional model concentrations from the CMAQ model, *C*_ADMS‐U‐Grid_ represents the ADMS‐Urban modeled concentrations with evenly distributed grid emissions of the regional model, and *C*_ADMS‐U‐Explicit_ indicates the ADMS‐Urban modeled concentrations with explicit high‐resolution emissions (e.g., 10 m). This equation explicitly states that concentrations arising from the short‐time scale dispersion of emissions within the regional model are subtracted from the total regional model concentrations, to be replaced with an explicit representation of these sources. Thus, the system does not double count emissions. More details could be found in our previous publications (Hood et al., 2018; Stocker et al., 2012). The local modeling of road sources in ADMS‐Urban can include street canyon effects, which affect the predicted concentrations both inside and outside a canyon (Hood et al., 2021). In the ADMS model, the H/W ratio is considered in street canyons, where a street is flanked by buildings on both sides to form a canyon‐like environment. The H/W ratio is defined as the average building height on both sides of the street canyon divided by the distance between the two sides. The ADMS‐Urban street canyon module was designed to account for street canyons with higher H/W ratios than the popular Operational Street Pollution Model, which was developed for H/W ratios around one. Although the street canyon module in ADMS‐Urban is limited to the availability of building morphology, the ADMS‐Urban model demonstrates the near‐source concentration gradient, showing the concentration differences at locations near to the sources and far away from sources within the same regional model cells. Few studies have applied coupled regional and very high‐resolution (street level) modeling techniques to investigate the traffic and industrial contributions to complex coupled O_3_ and PM_2.5_ issues through testing of emissions scenarios. A street‐scale model that provides a detailed representation of the spatial variation of pollutant gradients has clear advantages in terms of calculating health exposure over previous studies that have applied regional or global models.

Although urban‐scale models have been used to investigate air pollution interactions in megacities, very few‐coupled model systems based on ADMS‐Urban and CMAQ models have been applied in the metropolitan region of the Guangdong‐Hong Kong‐Macau Greater Bay Area (GBA). Zhang et al. (2015) integrated the CMAQ model with the CALifornia PUFF (CALPUFF) model to simulate the contribution of SO_2_ concentrations from local emissions in the GBA region. A regional European Monitoring and Evaluation Program Unified Model for the UK (EMEP4UK) with a resolution of 5 km was coupled with ADMS‐Urban to perform street‐level air pollutant simulations in London (Hood et al., 2018). Although ADMS‐Urban was applied within the Sixth‐Ring Road area in Beijing to simulate various pollutants (Biggart et al., 2020), it was not coupled with a regional model; instead, the background concentration levels were adapted directly from measurement data and were assumed to be distributed uniformly across the model domain. As a result, our study is the first localized regional‐to‐local scale model system that couples ADMS‐Urban and CMAQ to assess the sensitivity of two pollutants to emissions from traffic and industrial sectors in the GBA. The motivation for targeting these two sectors is related to the importance of the O_3_ precursors, VOC and NO_x_, derived from the anthropogenic industrial and traffic sectors in the GBA, respectively. Consequently, assessing the impact of changes to emissions of NO_x_ (and hence NO_2_) and VOCs is of particular interest. Section 2 of this paper describes the regional and local street‐scale model configurations and the sensitivity scenarios. The regional and local model simulation results and the model performance of selected monitoring stations are discussed in Section 3. Section 4 presents a discussion of the research findings, followed by the conclusions in Section 5.

## 2 Research Methods

### 2.1 Model Configuration

Figure S1 in Supporting Information S1 shows a research framework for the CMAQ–ADMS‐Urban air quality modeling system. The street‐scale resolution ADMS‐Urban dispersion model was coupled with the regional CMAQ model using the ADMS‐Urban Regional Model Link (ADMS‐Urban RML) to investigate O_3_ and PM_2.5_ concentrations and the sensitivity of both pollutants to emissions from the traffic and industrial sectors. The ADMS‐Urban RML is used to automatically prepare nested data from the regional model (CMAQ) and the mesoscale meteorological model (WRF) for the street‐level ADMS‐Urban model. ADMS‐Urban inherits the concentration outputs from the CMAQ model as background concentrations at each modeled hour. Most of the slow reactions are considered by the CMAQ chemical reaction scheme. The ADMS‐Urban model is specialized in capturing the fine concentration gradient and rapid chemical reactions when emissions are released from pollution sources, such as in traffic settings. Over time, the concentration gradient lowers; the CMAQ model can simulate regional transport and the associated chemical reactions. In addition to the CMAQ output, the following reaction sets are calculated by the ADMS‐Urban model. For NO_x_–O_3_, the Generic Reaction Set (Malkin et al., 2016) is used with an extra reaction introduced, that is, 2NO + O_2_ => 2NO_2_. For sulfates, sulfur dioxide is oxidized to particulates via the reactions 2SO_2_ + O_2_ => 2SO_3_, SO_3_ + H_2_O => H_2_SO_4_, and H_2_SO_4_ + 2NH_3_ => (NH_4_)_2_SO_4_. The Generic Reaction Set includes the following chemical reactions: ROC + hv => RP + ROC, RP + NO => NO_2_, NO_2_ + hv => NO + O_3_, NO + O_3_ => NO_2_, RP + RP => RP, RP + NO_2_ => SGN, RP + NO_2_ => SNGN, where the ROC means the reactive organic compounds, RP represents the radical pool, SGN is short for stable gaseous nitrogen product, and SNGN represents the stable nongaseous nitrogen product (Malkin et al., 2016).

The regional CMAQ model applied in this study is the same as that used to assess holistic emission control policies (Zhang et al., 2020), combined health effects (Zhang, Fung, Lau, Hossain, et al., 2021), and data assimilation of model bias corrections (Zhang, Fung, Lau, Zhang, & Huang, 2021) in our previous publications. In terms of the regional model configuration, detailed settings are described in the aforementioned publications, and only key points are listed below. The Sparse Matrix Operations Kernels Emissions (SMOKE) model was used to process the localized bottom‐up emission inventory, including industrial sources, mobile sources, power plants, residential sources, and marine sources in the GBA (Hong Kong Environmental Protection Department, 2019). The marine emissions were split into ocean‐going vessels, local vessels, and river vessels and were calculated using automatic identification system data. The emission inventory outside the GBA was adapted from Multi‐resolution Emission (MEIC) data (Tong et al., 2020). As shown in Figure S2 in Supporting Information S1, four nested domains with resolutions of 27 km (D1), 9 km (D2), 3 km (D3), and 1 km (D4) were utilized for the regional CMAQ model, and Domain 5 (6 km × 6 km), in an urban area of Guangzhou City, was chosen to drive the street‐level ADMS‐Urban model. The GBA includes Hong Kong (HK), Macau (MC), and the Pearl River Delta Economic Zone (PRD EZ), which includes nine cities; that is, Guangzhou (GZ), Shenzhen (SZ), Foshan (FS), Dongguan (DG), Zhuhai (ZH), Zhongshan (ZS), Jiangmen (JM), Huizhou (HZ), and Zhaoqing (ZQ). The WRF domains are larger than the CMAQ domain by at least 3–5 grids to remove the boundary effects of the WRF model on the CMAQ model. The boundary conditions of the outermost D1 domain were obtained from a global chemical transport model GEOS‐chem (Lam & Fu, 2010); boundary conditions for the remaining nested domains D2–D4 were obtained from the respective mother domains. Outputs from the SMOKE and CMAQ models were used to drive the ADMS‐Urban model.

ADMS‐Urban is a street‐scale resolution, quasi‐Gaussian plume dispersion model from the ADMS family, which has been widely applied worldwide to assess environmental impacts, mitigation strategies, and pollution concentration forecasts (Biggart et al., 2020; Carruthers et al., 1994; Hood et al., 2018; Lao & Teixidó, 2011). The model simulates the dispersion of pollutant emissions in urban areas by representing sources at high spatial resolution (primarily traffic and industry); modeling the influence of urban morphology on dispersion processes (street canyons, building density, tunnels, and road elevation) and applying simplified near‐field chemical schemes. Sharp concentration gradients resulting from emissions released from sources such as traffic can be resolved in the model calculations and captured for output using the irregularly spaced receptor grid generated by the model. Spatially variable meteorological parameters from the WRF model, such as wind and surface sensible heat flux, have been used as inputs for the ADMS‐Urban model to drive pollutant dispersion. “Background” pollutant concentrations, representing long‐range pollutant transport, have been derived from the CMAQ model hourly simulation data. Owing to a lack of representative source parameters (stack heights, efflux parameters), industrial and power plant sources were coarsely represented in the regional model using appropriate factors to disaggregate emissions vertically, whereas an explicit road network was applied to distribute the ground‐level traffic emissions in the ADMS‐Urban model. Two sets of explicit traffic emissions were prepared: one set replicated the CMAQ grid concentrations that were distributed evenly across the traffic emissions model grid and extracted from the CMAQ model grids; the second set redistributed the CMAQ grid traffic emissions into explicit high‐resolution traffic emissions within the facilitated road network. The gridded ADMS‐Urban concentrations using the evenly distributed grid emissions were reduced from the ultimate ADMS‐Urban concentration calculations to avoid double‐counting of emissions. The allocation formula for redistributing the traffic emissions is detailed in Biggart et al. (2020). The road length was calculated on the basis of the CMAQ grids. Different weighting factors were given to different types of roads, but the same factors were assigned to different air pollutants. The road network data were obtained from the OpenStreetMap source (OpenStreet Map Contributors, 2021), with some minor roads removed to reduce the computational costs. Urban morphology data, such as street canyon and building data, are lacking in the Guangzhou region; these could be considered in the coupled model in the future if such data become available. Detailed descriptions of the methodology of coupling a regional model with ADMS‐Urban are provided in previous publications (Hood et al., 2018; Stocker et al., 2012).

### 2.2 Sensitivity Scenario Design

The control measures affecting emissions from the traffic and industrial sectors were applied in the regional CMAQ model over the PRD EZ, and explicit traffic emissions scenarios were applied to the road traffic network modeled in ADMS‐Urban for the Guangzhou urban area. The provincial government in mainland China focuses largely on how the concentrations of air pollutants change if control measures are implemented in the PRD EZ; therefore, we assumed that there were no changes in emissions for HK and MC. The aim of the scenario was to investigate the concentration responses of local control measures. Four potential sensitivity scenarios were designed. As shown in Table 1, the Base case was a business as usual (BAU) scenario for both the regional CMAQ and local ADMS‐Urban models. Evaluation of the system was performed for a historical period with readily available measurement data. Meteorological conditions influence the likelihood of O_3_ episodes. As higher concentrations were recorded in the spring and autumn of 2019, O_3_ episodes in April and May 2019 were chosen for the control measures scenario. Table 1 lists three control scenarios. As NO_x_ and VOC are important precursors for O_3_ formation, the nonlinear relationship between the precursors and O_3_ is of great importance. Because of the short lifetime of NO_x_, which is emitted mainly from the traffic sector, the half‐traffic case considers a 50% reduction in traffic emissions for all standard pollutants in the coupled modeling system. As the majority of anthropogenic VOC emissions come from the industrial sector, the half‐industrial VOC case considers a 50% reduction in industrial VOC emissions only in the regional model, with BAU in the local model. Both control case integrates the control measures in both the half‐traffic and half‐industrial VOC scenarios.

**Table 1:** Scenario Design for the CMAQ–ADMS‐Urban Coupling System Integrating the Regional CMAQ Model and Local Street‐Level ADMS‐Urban Model

Scenarios	I. Base case	II. Half‐traffic case	III. Half‐industry VOC case	IV. Both control case
Scenario description	Business As Usual (BAU)	50% reduction in traffic emissions	50% reduction in industrial VOC emissions	Scenarios II & III
Regional CMAQ model emissions	BAU	50% emission reduction in Mobile sector (all pollutants)	50% emission reduction in VOC from Industrial sector	50% emission reduction in a) mobile sector (all pollutants) and b) VOC emissions from the industrial sector
Local street‐level model emissions	BAU	50% reduction in emissions from explicitly defined road traffic sources	BAU	50% reduction in emissions from explicitly defined road traffic sources

### 2.3 Scenario Emissions Comparison

It is important to place the “50% reduction” control measures in the context of total emissions. The main reason for halving the precursor emissions was to assess the sensitivity of the air pollutant concentrations to corresponding changes in emissions. The brute‐force method (also known as the zero‐out method, evaluating the concentration differences of two parallel air quality modeling runs with full emissions and a targeting reduced emission source [Thunis et al., 2019]) used in the sensitivity analysis would cause accuracy issues if small emission changes were applied (Clappier et al., 2017; Yarwood et al., 2007). A previous study (Tsimpidi et al., 2008) utilized the same strategy to assess fine particulate matter changes corresponding to halved NO_x_ and VOC emissions in the United States. However, although PM_2.5_ concentrations were investigated in different regions, no analyses were conducted for specific sectors. Therefore, halving the precursor emissions in sensitivity analyses of air quality modeling is a typical and effective way to evaluate the sectoral concentration responses. A summary of the total annual anthropogenic NO_x_, VOC, and PM_2.5_ emissions for the regional model domain covering the central GBA is presented in Figure S3 in Supporting Information S1. Both the “point” and “area” emissions categories are considered to represent primarily industrial activities, with VOC emissions affected by the “half‐industrial VOC” control. Relative to total emissions, the maximum reduction in NO_x_ is ∼17%, and that for PM_2.5_ is only 5%. For VOCs, which are impacted by both control measures in traffic and industrial sectors, emissions are reduced by a much larger amount, 47%. Although the proportions of biogenic and anthropogenic VOC may vary in different seasons and environmental conditions (e.g., temperature, humidity), a previous study estimated that biogenic emissions represent nearly 50% of China's total VOC emissions (Cao et al., 2018). Therefore, biogenic emissions are likely to contribute substantially to VOC emissions in the GBA. As a result, the maximum VOC reduction due to control measures is likely to be closer to 25%. In terms of a reduction in traffic emissions, the modeled scenario corresponds to a reduction in vehicle numbers and/or driving distances, rather than to improvements in vehicle technologies. Although technological improvements (including the introduction of electric vehicles) may reduce vehicle exhaust emissions to zero, nonexhaust particulate emissions, such as brake and tire wear, are a direct result of vehicle activity. Consequently, nonexhaust vehicle emissions are not mitigated by improvements to engine technology, although there may be associated technological improvements in relation to nonexhaust emissions, such as regenerative braking. Daily column emissions comparisons for NO_x_, VOC, and PM_2.5_ are provided in the Supporting Information S1 (Figures S3–S9). We assumed no emissions control activities in HK, as this study focuses on evaluating how air pollution in the PRD EZ changes in response to local controls.

## 3 Results

The regional model was configured and run for April 1–31 May 2019. The ADMS‐Urban model results were generated for the same period for the urban subdomains developed as a demonstration area for this study; that is, a 6‐km × 6‐km area in central Guangzhou. Period‐averaged concentrations were calculated. Both urban and rural locations were selected to illustrate variations in pollution. The period‐averaged prediction is the hourly average value for the entire modeling period (April 1–31 May 2019). This period was chosen owing to the occurrence of frequent O_3_ episodes, and the local government is particularly interested in exploring the mechanisms of occurrence in the Guangzhou urban area. A 1‐week spin‐up period was used.

Statistical parameter performances (Table 2) and time series plots for typical monitoring stations (Figures S10–S12 in Supporting Information S1) in the GBA (China National Environmental Monitoring Center, 2021; Hong Kong Environmental Protection Department, 2021) were analyzed to validate the base case of the regional CMAQ model at a 1‐km resolution. Table 2 clearly shows that the CMAQ model obtains an acceptable level of accuracy for PM_2.5_ simulations (with mean fractional bias ≤ ±0.6 and mean fractional error ≤0.75) according to the criteria proposed by Hu et al. (2016). The averaged O_3_ observation is 28.6 ppb, and the mean O_3_ simulation is 28.9 ppb, with an Index of Agreement (IOA) of 0.63. Although the CMAQ model underestimates NO_2_ concentrations by 2.5 ppb, the IOA of NO_2_ is up to 0.57, and the root‐mean‐square error is around 10. Overall, the CMAQ model simulation is considered an acceptable input to drive the ADMS‐Urban model. In addition to the capability of the CMAQ model to capture the main trend in the time series plots during the modeling period, Figures S13–S15 in Supporting Information S1 show the time series comparisons of the base case for both the CMAQ and ADMS‐Urban models. Substantial improvement was observed during specific pollution episodes, which illustrates the advantages of coupled urban dispersion models. Table 3 shows the statistical performance of the base scenario for CMAQ and ADMS‐Urban models in the Guangzhou urban region (domain 5), compared with the monitoring observational data (China National Environmental Monitoring Center, 2021). The statistic parameters shown in bold and italics indicate the best performance based on criteria proposed by Hood et al. (2021) to evaluate the ADMS‐Urban model performance. The model simulations have improved performance in the ADMS‐Urban model with lower model biases compared with the CMAQ model. Among the three pollutants, the ADMS‐Urban model was improved the most substantially in simulating NO_2_ concentrations with higher IOA (0.6), lower Fractional bias (−0.130) and Normalized Mean‐Square Error (0.265), and a greater proportion of modeled concentrations within the factor of two of observed concentrations (Hood et al., 2021). It highlights the strength of resolving sharp concentration gradients near the roads by the ADMS‐Urban model.

**Table 2:** Statistical Performance of the CMAQ Base Scenario in Regional Model Domain 4, at a Resolution of 1 km

	OBS	Model	IOA	RMSE	MNB	MNE	MFB	MFE
NO2	15.3	12.8	0.57	10.37	0.26	0.81	−0.19	0.61
O3	28.6	28.9	0.63	18.51	1.32	1.63	0.18	0.61
PM2.5	18.2	13.92	0.49	12.03	0.07	0.65	−0.25	0.57

*Note.* The units of NO_2_ and O_3_ are ppb, the unit of PM_2.5_ is μg/m^3^. The statistical parameters include averaged hourly observational data (OBS), averaged hourly model simulations (Model), Index of Agreement (IOA), Root‐Mean‐Square Error (RMSE), Mean Normalized Bias (MNB), Mean Normalized Error (MNE), Mean Fractional Bias (MFB), and Mean Fractional Error (MFE).

**Table 3:** Statistical Performance of the Base Scenario for CMAQ and ADMS‐Urban in Guangzhou Domain 5

		OBS	Model	IOA	Fb	NMSE	Fac2
NO2	CMAQ	43.9	31.6	0.56	−0.326	0.405	0.69
	ADMS	43.9	***38.7***	***0.60***	***−0.130***	***0.265***	***0.82***
O3	CMAQ	43.4	52.1	0.78	0.185	0.352	0.54
	ADMS	43.4	***48.4***	0.78	***0.111***	0.364	0.53
PM2.5	CMAQ	24.0	21.8	0.46	−0.099	0.397	0.72
	ADMS	24.0	***24.2***	***0.47***	***0.005***	***0.340***	***0.75***

*Note.* The units of NO_2_ and O_3_ are ppb, the unit of PM_2.5_ is μg/m^3^. The bold and italic values highlight the improved ADMS simulations comparied with CMAQ model. The statistical parameters include averaged hourly observational data (OBS), averaged hourly model simulations (Model), Index of Agreement (IOA), Fractional bias (Fb), Normalized Mean‐Square Error (NMSE), and Fraction of modeled hourly concentrations within a factor of two of observations (Fac2).

### 3.1 Regional Model Period‐Averaged Air Quality Maps

**Figure 1:**
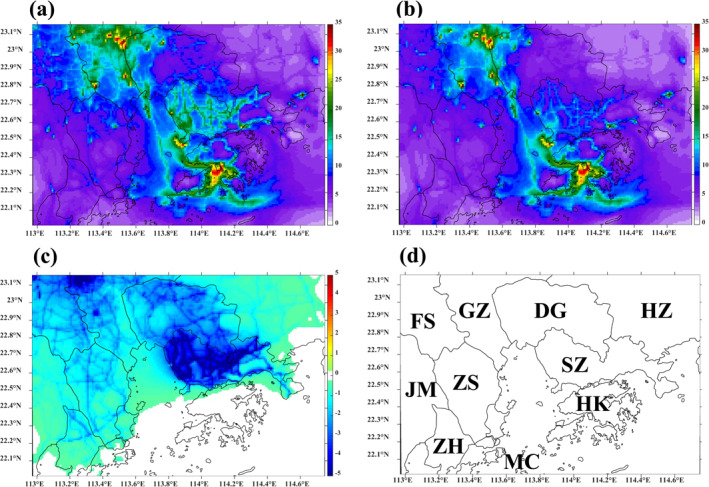
Simulated spatial maps of period‐averaged NO_2_ concentrations from the Community Multiscale Air Quality (CMAQ) model for (a) Base case, (b) Half‐traffic case, and (c) Difference plot: Both controls – Base case (ppb). (d) Major cities in Domain 4: Guangzhou (GZ), Shenzhen (SZ), Foshan (FS), Dongguan (DG), Zhuhai (ZH), Zhongshan (ZS), Jiangmen (JM), Huizhou (HZ), Hong Kong (HK), and Macau (MC).

The regional CMAQ model was run for two typical months for the base case and the three sensitivity scenarios to drive the respective ADMS‐Urban base case and corresponding sensitivity scenarios. The spatial concentration maps of the differences among the scenarios show the concentration changes due to the designed halved emissions in different sectors. Figure 1 shows the simulated spatial concentration maps of period‐averaged NO_2_ concentrations from the regional CMAQ model, in which the half‐traffic case dominants by a substantial margin. Therefore, the traffic sector‐related scenarios are selected to demonstrate. Figures 1a and 1b display the period‐averaged NO_2_ concentrations for major PRD EZ cities for the base case and half‐traffic case, respectively, at a 1‐km grid resolution. As expected, there were clear reductions in NO_2_ in Guangzhou and Shenzhen, especially for the road network, owing to the implementation of increased controls on emissions from the traffic sector in these two cities; the traffic sector is the dominant source of NO_2_. In terms of the spatial distributions illustrated in Figures 1a and 1b, NO_2_ concentrations are markedly higher in HK (south of Shenzhen), in industrial areas toward Guangzhou, and along shipping lanes than in the urban area in the GBA. Figure 1c quantifies the reductions in NO_2_ concentrations for the modeling period, which are as large as 5 ppb in central Guangzhou and Shenzhen.

**Figure 2:**
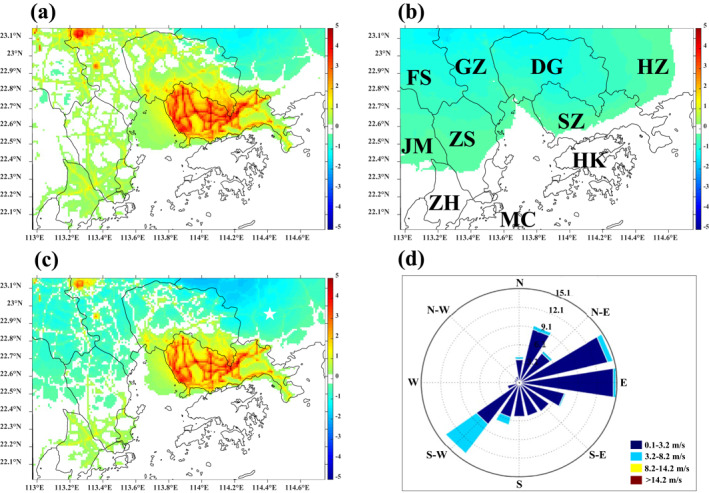
Simulated spatial maps of period‐averaged O_3_ concentrations ‐ Difference plots from the Community Multiscale Air Quality (CMAQ) model for (a) Half‐traffic – Base case; (b) Half‐industry VOC – Base case, and (c) Both controls – Base case (ppb); (d) Wind Rose diagram showing regional wind directions. Major cities in Domain 4: Guangzhou (GZ), Shenzhen (SZ), Foshan (FS), Dongguan (DG), Zhuhai (ZH), Zhongshan (ZS), Jiangmen (JM), Huizhou (HZ), Hong Kong (HK), and Macau (MC).

Figure 2 shows the simulated spatial distribution maps of period‐average O_3_ concentrations from the CMAQ model for major PRD EZ cities in the (a) Half‐traffic minus Base case; (b) Half‐industrial VOC minus Base case, (c) Both controls minus Base case; and (d) Wind Rose diagram showing regional wind directions. The Red shading indicates worsening O_3_ concentrations, and blue indicates improving conditions. Figure 2a relates to the effectiveness of the half‐traffic case. Owing to the substantial reduction in NO_x_ concentrations (Figure 1c), the half‐traffic scenario leads to significant increases in O_3_ concentrations in Guangzhou and Shenzhen, mainly from the traffic sector. A slight increase (>1 ppb) is observed in other areas, aside from upwind rural areas where an improvement (around 1 ppb) is noted. When the half‐traffic measures are implemented, the NO_x_ concentrations drop substantially, leading to less NO_x_ titration. Therefore, worsening O_3_ concentrations are observed in urban areas of Guangzhou and in Shenzhen, especially near road networks. This phenomenon suggests a VOC‐limited O_3_ formation regime in Guangzhou, which is consistent with the results of previous studies (Wang, Lyu, et al., 2019; Zhang, Fung, Lau, Hossain, et al., 2021). Conversely, in the rural areas to the northeast of the domain, that is, downwind of the highly polluting areas in mainland China (outside the GBA), O_3_ concentrations decrease in response to the controls because lower levels of oxidants (the sum of NO_2_ and O_3_) are present in the atmosphere.

Figure 2b relates to the effectiveness of the half‐industrial VOC case on O_3_ concentrations. We observe that O_3_ concentrations are reduced throughout the domain. This verifies the importance of controlling VOC in areas susceptible to VOC‐limited O_3_ formation, such as Guangzhou and Shenzhen. The main reason for this observation is that the limited VOCs correspond to lower levels of RO_2_ radicals, causing excess NO to react with O_3_ in the atmosphere, resulting in less O_3_ being generated (Wang et al., 2017). During this process, NO_2_ concentrations drop owing to the decrease in RO_2_ radicals but then increase owing to the consumption of O_3_ by excess NO, producing more NO_2_. Therefore, the change in net NO_2_ would be minimal. Figure 2b also shows that O_3_ concentrations are transported from upwind areas, highlighting the impact of regional transport on O_3_ concentrations.

Figure 2c shows the contributions of the control cases in both the half‐traffic and half‐industrial VOC cases simultaneously. Although the O_3_ concentrations in urban regions still increase owing to NO_x_ controls, the magnitude of the increase is mitigated. In most locations outside the urban areas, the O_3_ concentrations are reduced. It is noteworthy that, in a large area of the domain (upper left locations in Figure 2c), the trend of O_3_ concentrations is inverted, changing from an increase in response to traffic controls to decreases in both control cases. This finding highlights the importance of coordinated controls for both traffic and industrial VOC sources. However, the trend in the downwind area (lower‐left corner of Figure 2c) remains positive, mainly because of the accumulation of transported O_3_, which indicates the long‐range transport of O_3_.

**Figure 3:**
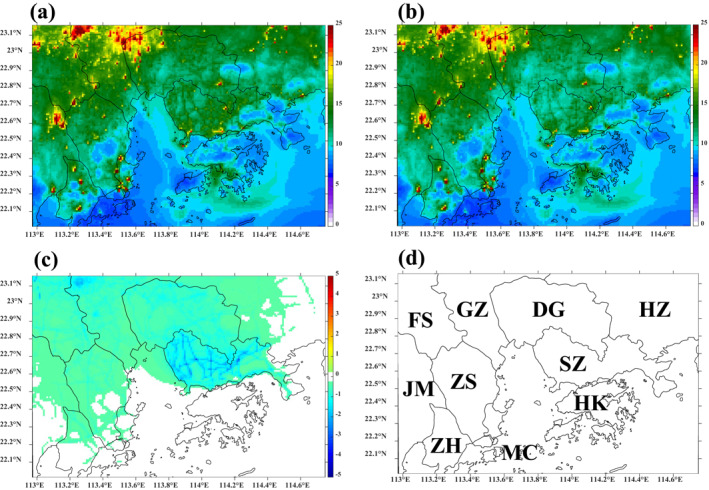
Simulated spatial maps of period‐averaged PM_2.5_ concentrations from the Community Multiscale Air Quality (CMAQ) model for (a) Base case, (b) Half‐traffic case, and (c) Difference plot: Both controls – Base case (μg/m³). (d) Major cities in Domain 4: Guangzhou (GZ), Shenzhen (SZ), Foshan (FS), Dongguan (DG), Zhuhai (ZH), Zhongshan (ZS), Jiangmen (JM), Huizhou (HZ), Hong Kong (HK), and Macau (MC).

Figure 3 shows the simulated spatial distribution maps of period‐averaged PM_2.5_ concentrations from the CMAQ model at a 1‐km resolution in the (a) Base case, (b) Half‐traffic case; and (c) Difference plot: Both controls minus Base case. Although the reduced total PM_2.5_ emission is ∼5% (Figure S3c in Supporting Information S1) and the reduced total NO_x_ emission is ∼10% (Figure S3a in Supporting Information S1) in the traffic sector, a noteworthy reduction (∼10%–15%) in PM_2.5_ concentrations is observed in the urban Guangzhou area and central Shenzhen, highlighting the strengthened traffic control measures implemented in mega‐cities. This is also consistent with our previous publication (Wu et al., 2013). We consider marine sources as being separate from traffic sources, which is why a little change is observed along shipping routes in the half‐traffic case. The half‐industrial VOC case has a small impact on PM_2.5_, which will be discussed under the ADMS‐Urban results (Section 3.2). Figure 3c in Supporting Information S1 shows that both control scenarios lead to a noteworthy reduction in PM_2.5_ concentrations across the mitigation domain, with reductions of up to 3 μg/m³ in central Shenzhen and Guangzhou, focused mainly on the road network.

### 3.2 Street‐Level Urban Model Variation of Air Quality Maps

**Figure 4:**
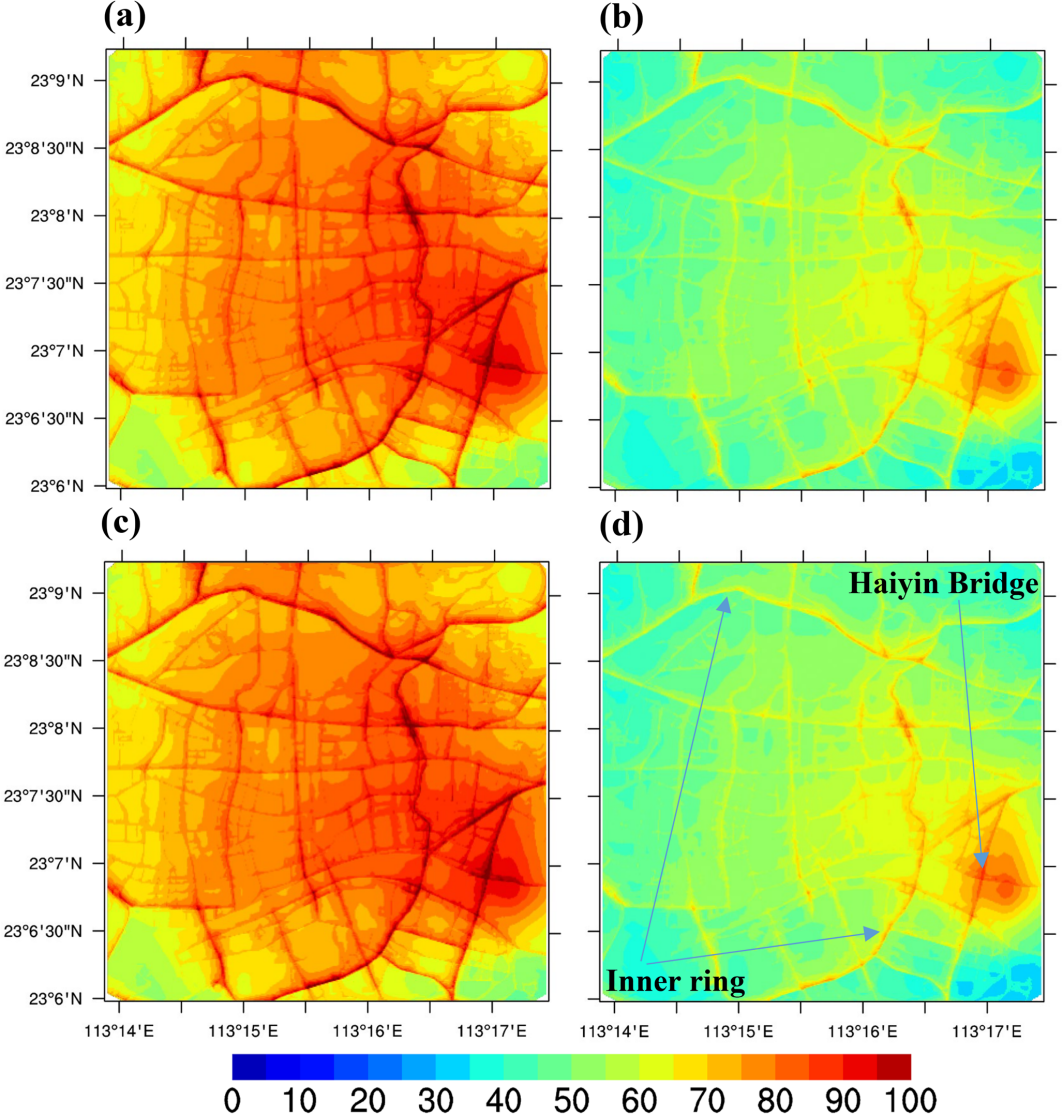
Simulated high‐resolution spatial maps of NO_2_ (Guangzhou domain) from the ADMS‐Urban model at 19:00, 28 May 2019 for (a) Base case, (b) Half‐traffic case, (c) Half‐industry VOC case, (d) Both control case (μg/m^3^).

Concentrations within the ADMS‐Urban system in the Guangzhou urban region were calculated at a high spatial resolution (<10 m), specifically for traffic emissions. Hourly concentrations were obtained rather than average concentrations over the 2 months, as was done for the regional CMAQ model domain. For some pollutants, the resultant detailed, hourly air quality maps are consistent with the metrics included in Chinese air quality standards (i.e., the 160 μg/m³ standards for O_3_ concentrations, which are applicable in urban areas). Figure 4 shows the simulated high‐resolution spatial distribution maps for NO_2_ (Guangzhou domain) from the ADMS‐Urban model in the: (a) Base case; (b) Half‐traffic case; (c) Half‐industrial VOC case; and (d) Both control cases during an afternoon in May. High concentrations of NO_2_ are clearly observed on the road network. The largest hot spot occurs near the inner ring road and Haiyin Bridge, where traffic is heavy during afternoon peak times. NO_x_ concentrations in the urban‐scale model are determined by emissions sources, pollution dispersion, chemical reactions, and background concentrations. NO_x_ has a short life cycle; therefore, it is derived mainly from local sources. The half‐traffic case in Figure 4b shows substantial decreases in NO_2_ concentrations throughout the urban domain owing to increasing traffic controls implemented in Guangzhou. The area with excessive NO_2_ is significantly reduced. However, local NO_2_ concentrations do not change with variations in the reduced industrial VOC cases (Figure 4c). This is mainly because the ADMS‐Urban model does not include explicitly industrial emissions, and less NO_2_ may be transported from the background regional CMAQ model. Therefore, the case with both controls (Figure 4d) is consistent with the reduced traffic case (Figure 4b).

**Figure 5:**
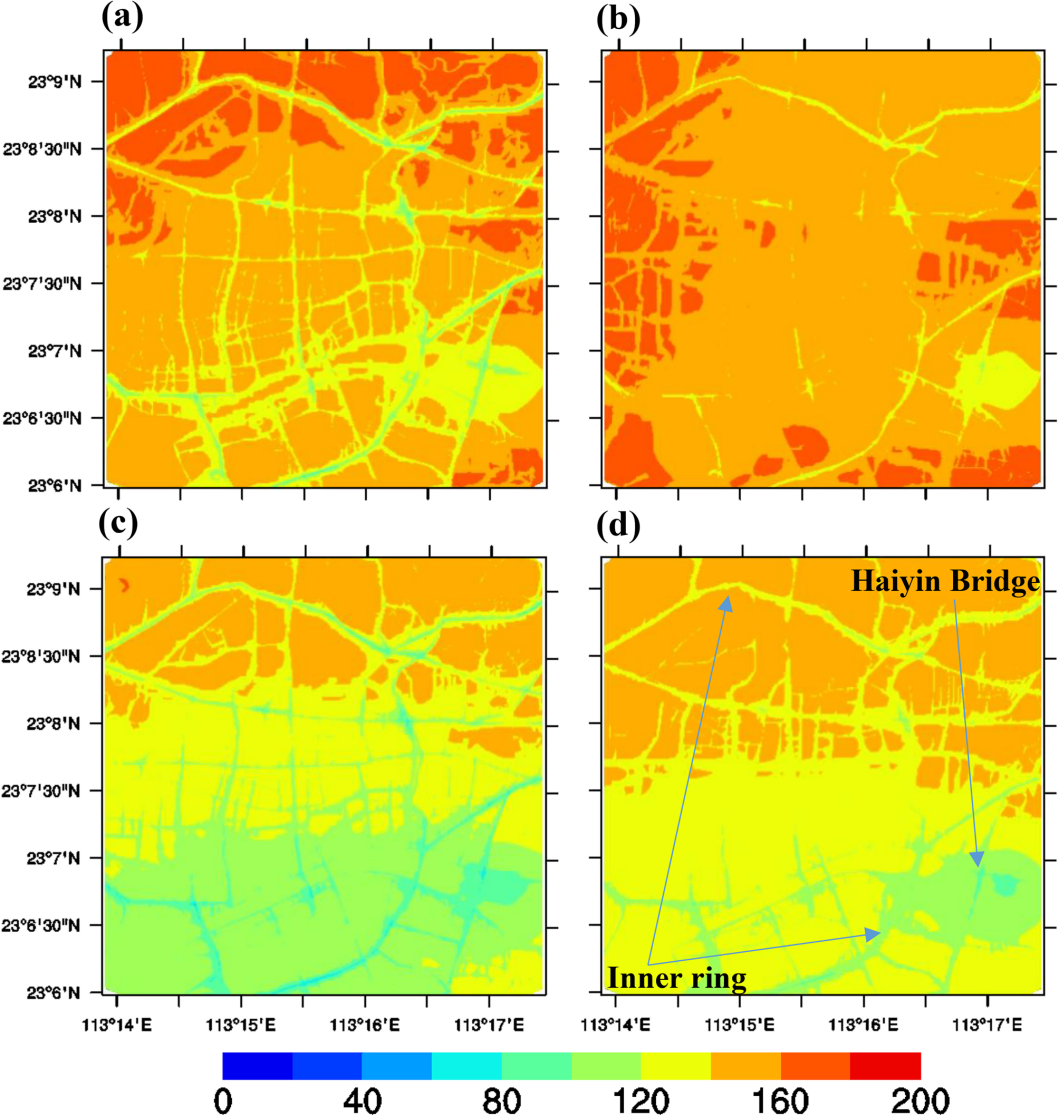
Simulated high‐resolution spatial maps of O_3_ (Guangzhou domain) from the ADMS‐Urban model at 14:00, 10 May 2019 for (a) Base case, (b) Half‐traffic case, (c) Half‐industry VOC case, (d) Both‐control case (μg/m^3^).

Figure 5 presents the simulated high‐resolution spatial distribution maps of O_3_ (Guangzhou domain) from the ADMS‐Urban model in the middle of the day during May for: (a) Base case; (b) Half‐traffic case; (c) Half‐industrial VOC case; (d) Both control case. For the road network, O_3_ concentrations are lower than NO_2_ concentrations. As indicated earlier in relation to the regional model results, reducing traffic emissions increases the spatial extent of excessive O_3_ in urban areas owing to reduced NO_x_ titration of O_3_ (Figure 5a vs. 5b). Conversely, reducing industrial VOCs leads to a reduction in the area of O_3_ exceedance within the local domain (Figure 5a vs. 5c). When both controls are applied in the local area (Figure 5a vs. 5d), the net effect is a slight increase in near‐road O_3_ concentrations but a decrease in concentrations elsewhere. This is an interesting result that again demonstrates the importance of accounting for both regional and local dispersion and chemistry.

**Figure 6:**
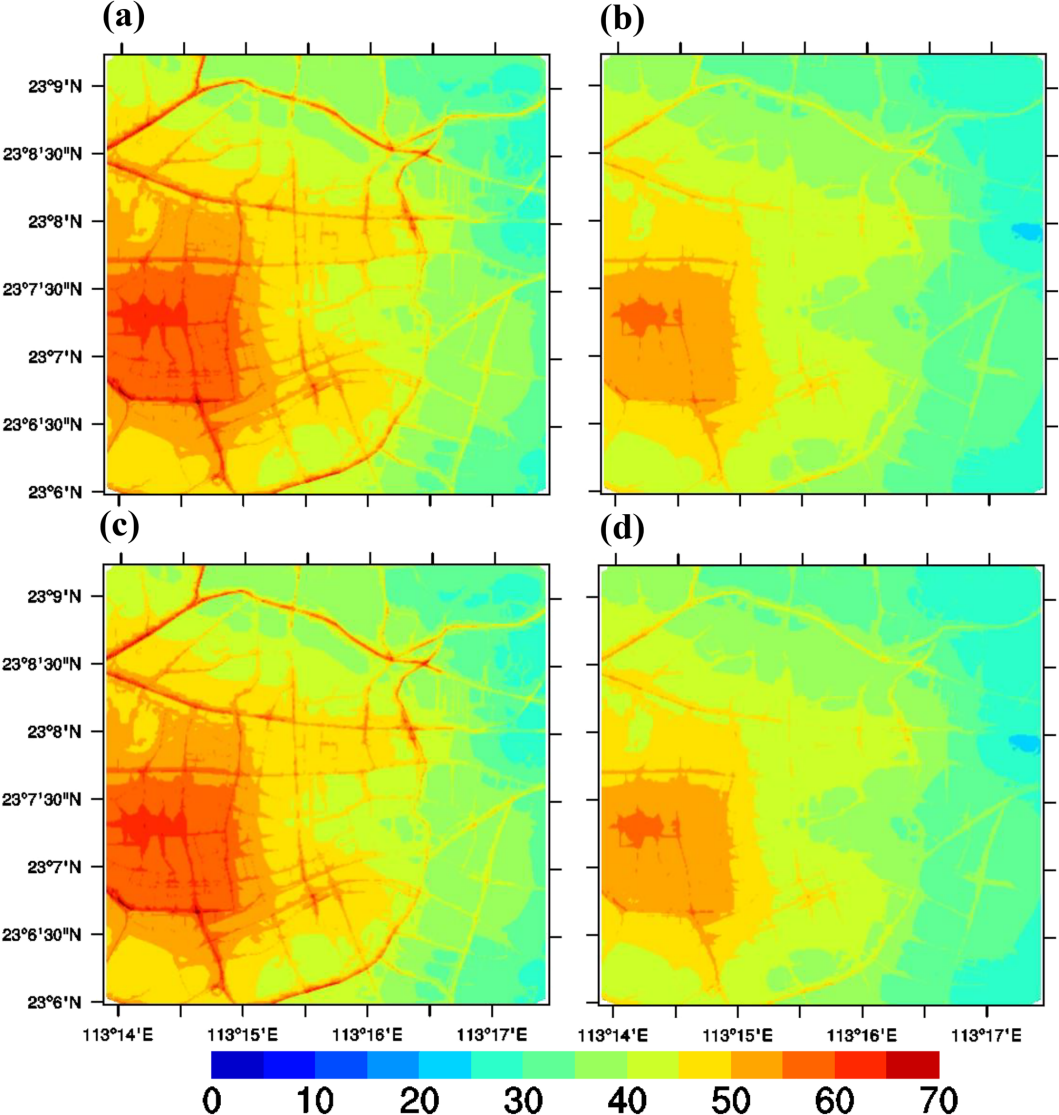
Simulated high‐resolution spatial maps of PM_2.5_ (Guangzhou domain) from the ADMS‐Urban model at 18:00, 17 April 2019 for (a) Base case, (b) Half‐traffic case, (c) Half‐industry VOC case, (d) Both‐control case (μg/m^3^).

The modeled PM_2.5_ concentrations reflect a different time of day, as the atmospheric conditions associated with PM_2.5_ pollution episodes differ from those associated with O_3_ and NO_2_. PM_2.5_ concentrations at 18:00 on 17 April 2019 are shown for all four scenarios in Figure 6. Although there is a very small relative reduction in PM_2.5_ emissions (Figure S3 in Supporting Information S1), the impact in urban areas is significant during this episode (Figures 6a vs. 6b), as this reduction relates to near‐ground traffic sources. The change in industrial VOC emissions has little effect (Figures 6a and 6c) on PM_2.5_ concentrations. In our scenario, the negative impacts on PM_2.5_ on reduced industrial VOC emissions are mainly observed in the background regional CMAQ model, as the ADMS‐Urban model has no explicit industrial sources in the reduced industrial VOC scenario. Reducing industrial VOC emissions will directly impact oxidant levels, thus impacting the formation of nitrate, sulphate, and secondary organic aerosols, which are important components of PM_2.5_. When VOC emissions are reduced by 50%, the level of oxidants in summer will increase, leading to increased sulphate or nitrate formation. However, organic matter will decrease due to the decreased secondary organic aerosol caused by decreased VOC emissions. Therefore, the net change in PM_2.5_ would be small. This result is consistent with a previous study (Tsimpidi et al., 2008), indicating that controlling industrial VOC emissions may not be an efficient method of controlling PM_2.5_. The simultaneous control of PM_2.5_ and O_3_ is a complex issue, and mitigation strategies will vary between areas with different formation regimes (i.e., VOC‐limited, NOx limited, or NH_3_‐rich/poor) (Xing et al., 2019). NH_3_ emissions need to be considered to further mitigate PM_2.5_ concentrations in the PRD EZ, as NH_3_ has also been detected in eastern China, as well (Geng et al., 2019).

### 3.3 Modeled Concentrations at Selected Urban and Rural Locations

**Figure 7:**
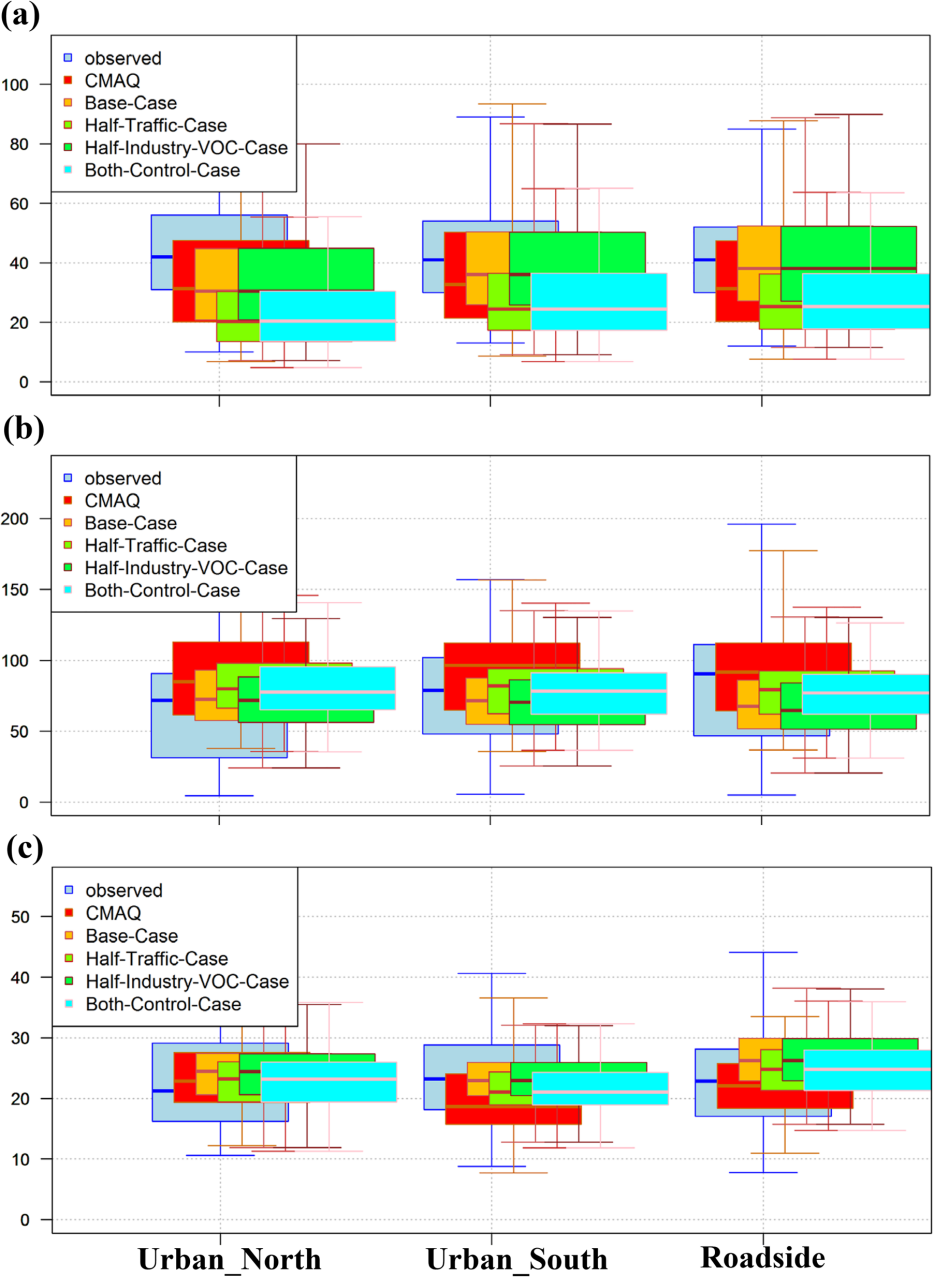
Box plots comparing measured concentrations (pale blue) and base regional Community Multiscale Air Quality (CMAQ) model concentrations (red) to the four high‐resolution coupled system model scenarios from ADMS‐Urban model for: Base case (orange), Half‐traffic case (light green), Half‐industry VOC case (darker green), and Both controls (bright blue) for (a) daily maximum hourly NO_2_, (b) daily maximum 8‐hr rolling O_3_, and (c) daily average PM_2.5._ Unit is in μg/m³.

This section discusses pointwise concentrations. Where possible, the locations considered relate to air quality measurement sites within the domain. Figure S2 in Supporting Information S1 shows the locations of the three reference monitors located within the coupled‐system urban model domain of Guangzhou; in addition to other pollutants, NO_2_, O_3_, and PM_2.5_ concentrations were recorded at these sites. Figure 7 compares the modeled concentrations to the measurements recorded at these three locations for the base case and three coupled model scenarios in addition to the base case regional model. Box plots of the short‐term pollutant metrics are shown, including the daily maximum hourly NO_2_, daily maximum 8‐hr rolling O_3_, and daily mean PM_2.5_. As this is the first time the regional model concentrations have been presented alongside the coupled model concentrations, it is worth noting the differences in the concentrations obtained using the two modeling approaches. Specifically, for NO_2_ and PM_2.5_ at most of the sites, the coupled system predicts higher concentrations than the relatively coarse resolution regional model; for O_3_, the coupled system predicts lower concentrations. These differences are expected at the monitoring locations, which are strongly influenced by local road traffic source increments. The respective concentration changes at the selected monitoring stations in the various sensitivity scenarios are similar to the trend illustrated in the comparisons of the spatial concentration map. Figure 7a shows that the NO_2_ concentrations are derived mainly from the traffic sector. The effects of NOx titration on the O_3_ concentration in Figure 7b drive up the O_3_ concentrations; therefore, reducing industrial VOC emissions sources is more effective for O_3_ control, revealing a VOC‐limited regime in this region.

In terms of the differences in modeled concentrations for the three scenarios, across all sites, the maximum decrease in the median NO_2_ hourly metric owing to emissions controls is >11 μg/m³ at the roadside site, which corresponds to the implementation of traffic controls. In terms of O_3_, the maximum increase in the median value is >10 μg/m³ for the half‐traffic scenario. However, this increase is reduced to <7 μg/m³ when both controls are applied simultaneously. The decrease in the median PM_2.5_ is <2 μg/m³ for the low‐traffic scenario.

**Figure 8:**
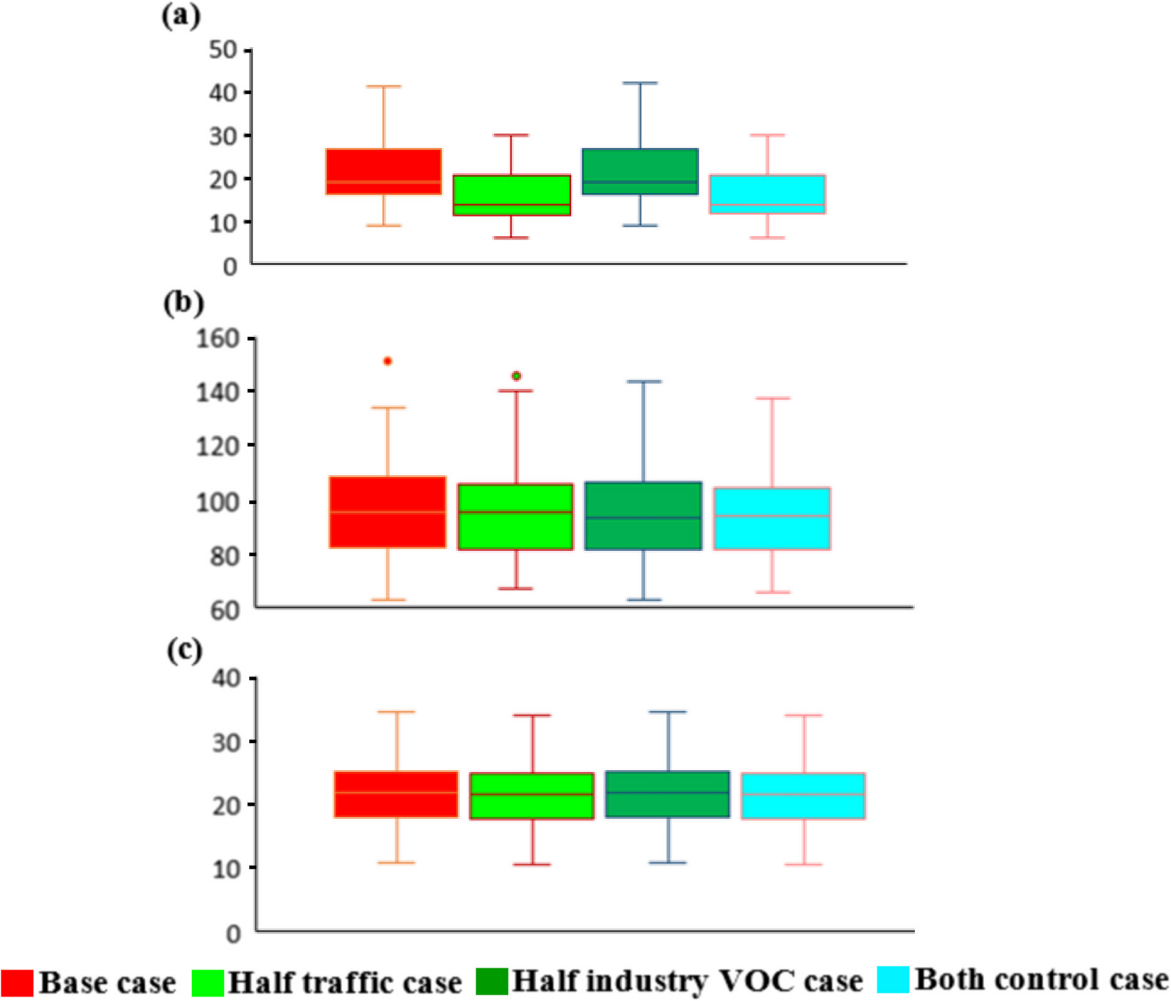
Box plots comparing the regional Community Multiscale Air Quality (CMAQ) model concentrations at a rural location (white star in Figure 2): Base case (red), Half‐traffic case (light green), Half‐industry VOC case (darker green), and Both controls (bright blue) for: (a) daily maximum hourly NO_2_, (b) daily maximum 8‐hr rolling O_3_, and (c) daily average PM_2.5._ Unit is in μg/m³.

It is of interest to quantify the decrease in O_3_ concentrations to the northeast of the model domain, as shown in Figure 2c. Unfortunately, data were unavailable for this rural location. Furthermore, the coupled system has only been configured for the example urban subdomains in Guangzhou. Consequently, the only comparison to be made at this location is between concentrations calculated by the regional model. Concentration data for the location indicated by the white star in Figure 2 are presented in Figure 8; the metrics calculated for NO_2_, O_3,_ and PM_2.5_ are the same as those presented in Figure 7. Of the three pollutants modeled at this rural location, the different emissions mitigation options only significantly alter the NO_2_ concentrations. This is unsurprising because Figure S3 in Supporting Information S1 shows that traffic emissions contribute a large proportion of the NO_x_ emissions over the whole domain, so changes to NO_x_ emissions are likely to impact NO_2_ concentrations in either rural or urban areas. Conversely, traffic makes up a relatively small proportion of primary PM_2.5_ emissions in rural areas, where ambient PM_2.5_ levels are more influenced by industrial point and area source emissions, in addition to the formation of secondary organic and inorganic particulate matter (Wu & Xie, 2018).

**Figure 9:**
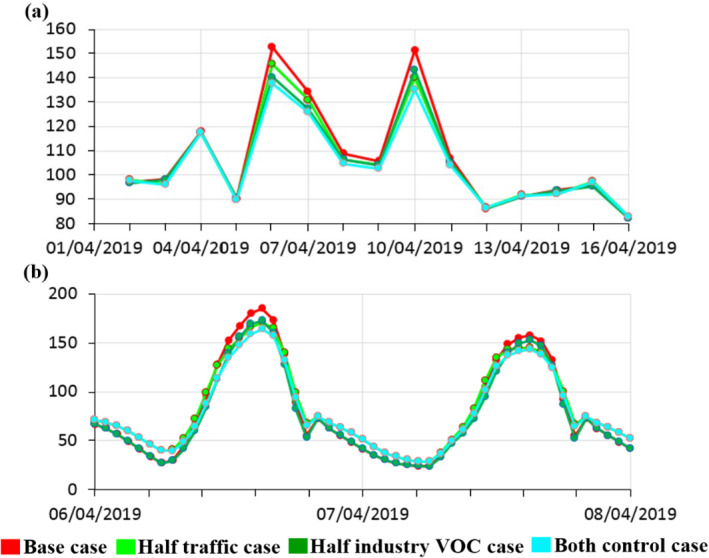
Regional Community Multiscale Air Quality (CMAQ) model predictions for (a) daily‐maximum 8‐hourly average O_3,_ (b) hourly average O_3_ during an episode in April 2019 at a rural location (white star in Figure 2) to the north‐east of the regional model domain: Base case (red), Half‐traffic case (light green), Half‐industry VOC case (darker green), and Both controls (bright blue). Unit is in μg/m^3^.

In terms of O_3_, there is a relatively minimal reduction in the median maximum 8‐hr averaged concentrations resulting from the reduced VOC emissions scenario. This is perhaps surprising when considering Figure 5 as, for the corresponding scenarios, decreases of tens of μg/m³ are shown throughout the urban model domain. To understand this, it is helpful to look at a time series of modeled O_3_ concentrations during an episode (Figure 9a). Here, we see that although there usually is very little difference in concentrations, the mitigation scenarios have a substantial impact in this rural location when O_3_ levels are at their highest (up to 15 μg/m³ for the 8‐hr rolling average metric) and with greater hourly concentration differences (up to 25 μg/m³) over the same period.

## 4 Discussion

A regional‐to‐local scale coupled modeling system consisting of a regional CMAQ model (Zhang et al., 2020) and a street‐scale ADMS‐Urban model (Biggart et al., 2020) was implemented to explore the sensitivity of NO_2_, O_3_, and PM_2.5_ to controls on traffic sources and industrial VOC emissions in the GBA, at varied resolutions. The high‐resolution concentration gradients (<10 m) have been cautiously resolved (Figures 4, 5, 6) using specific traffic emissions, which aid the assessment of health impacts in densely populated urban regions (Schmitz et al., 2019). A growing number of studies using satellite instruments, land‐use regression models, or directly measured personal exposure have been conducted to obtain high‐resolution spatial air pollution concentration maps (Apte et al., 2017), highlighting the importance of high‐resolution (10–20 m) spatial data sets. Our coupled modeling system makes use of a coarse regional model that provides time‐varying background concentrations to drive street‐level air dispersion models such as ADMS‐Urban, which specializes in capturing rapid chemical reactions. The initial results of the presented Guangzhou case are promising, providing more information on NO_x_–O_3_ sensitivity that is consistent with findings from previous modeling studies (Cheng et al., 2021; Ma et al., 2021; Wu et al., 2021; Zhang, Fung, Lau, Hossain, et al., 2021). This finding indicates that synergistic controls of NO_x_ and VOCs are promising means for the simultaneous mitigation of PM_2.5_ and O_3_, consistent with the findings of Wu et al. (2021). By coupling the regional‐to‐local scale model, the background concentration fields play a key role in the spatial variations of urban modeling simulations (Figures 4c, 5c and 6c). Further efforts to refine emissions in both the regional and urban‐scale models are necessary, especially for more explicit emissions sectors.

More stringent controls on industrial VOC emissions are found to be essential for inverting O_3_ concentrations from a worsening trend to a slight improvement (Figures 2b and 5c). This finding sheds light on the importance of applying stringent VOC control measures to the industrial sector in the near future. Previous studies (Chen et al., 2019; Mozaffar et al., 2020) have shown the efficiency of O_3_ controls in response to different VOC/NO_x_ ratios and varying O_3_ formation regimes. The effects of reducing these ratios should be further explored from a high‐resolution perspective in light of the substantial influence of biogenic VOC emissions on climate change (Li et al., 2018).

Urban/rural locations were selected for the analysis of the relative changes in the metric of different pollutants during pollution episodes. The magnitude of these relative changes should be taken in the context of the metric considered: hourly values (i.e., NO_2_) demonstrate the greatest variations because the maximum differences at peak traffic times are quantified; conversely, for daily averaged values (i.e., for PM_2.5_), the impact of peak values is smoothed out by the inclusion of hours where pollutant concentrations may be dominated by regional rather than local air pollution. O_3_ episodes are currently of particular interest to government officials and stakeholders. Our modeling work has demonstrated while O_3_ concentrations increase in urban areas as a result of the mitigation options considered, O_3_ concentrations decrease in upwind areas. Inspection of the modeled pollutant concentrations at a rural location to the northeast of the modeling domain during an O_3_ episode shows that the concentrations were reduced by up to 15 μg/m³ for the 8‐hr metric and up to 25 μg/m³ for the 1‐hr metric.

Although the implemented coupled CMAQ–ADMS‐Urban modeling system is capable of resolving the fine concentration gradient near road networks in this study, several limitations remain to be further investigated in future studies. First, more complete emission sectors, such as point, industry, or residential sources, should be included to construct holistic, high‐resolution concentration maps. Second, the urban domain should be further expanded to cover the whole GBA to obtain more complete measurements for model validation and exploration of photochemical mechanisms. Finally, the street canyon module and more detailed building morphology will most certainly benefit accurate calculations of the dispersion of air pollutants.

ADMS‐Urban resolves the dispersion and chemical processes that occur in the close proximity to road sources (a few meters). The magnitude of NO_x_ and NO_2_ concentrations close to roads are not only strongly influenced by traffic volumes and driver behavior (via emission rates) but also the building morphology (Hood et al., 2021) and city infrastructures such as road elevation (O'Neill et al., 2021) and tunnels. However, although the building/infrastructure features influence dispersion processes in the near‐field, concentrations a few hundred meters downwind of a source are broadly unaffected, which is demonstrated through the use of regional chemical transport models that are configured to provide accurate predictions of urban background concentrations without considering detailed urban morphology. Particulate matter and ozone concentrations are governed by regional dispersion and chemistry processes, so they are less influenced by near‐field building effects.

The inclusion of building and other urban morphology in an ADMS‐Urban model setup is highly recommended, where data are available. The use of urban morphological data will improve pollutant concentration predictions in urban areas; where street canyons are not modeled, in‐canyon concentrations of NO_2_ and NO_x_ are likely to be underpredicted by the model, and urban background concentrations may be slightly overpredicted (relating to the influence of buildings elevating emissions above the building canopy). However, for this study, buildings data were unavailable.

## 5 Conclusion

To address the challenges of controlling PM_2.5_ and O_3_ concentrations simultaneously using an ultrahigh spatial resolution approach, this study presents the regional air quality CMAQ model coupled to the street‐scale ADMS‐Urban model. This coupled system allows a thorough assessment of the impacts of halved traffic emissions and industrial VOC emissions on ambient NO_2_, O_3_, and PM_2.5_ concentrations, creating a holistic representation of pollution mitigation at a range of spatial resolutions and highlighting the interactions between emissions, meteorological conditions, and O_3_ concentrations. Both the regional and urban‐scale models illustrate the VOC‐limited O_3_ formation regime in Guangzhou and highlight the importance of synergistic control of NOx and VOC for mitigating O_3_ and PM_2.5_ pollution, especially with regard to strengthening controls on industrial VOC sources. With coupling, the street‐scale ADMS‐Urban model resolves the sharp concentration gradients in the vicinity of road sources. Urban and rural locations in central Guangzhou are used as examples to better interpret the findings, which will be beneficial for government policymaking.

Although the detailed mitigation pathways modeled here support the second phase of the Air Pollution Prevention and Control Action Plan—the Three‐Year Action Plan for Clean Air—released by the State Council of China in 2018, further refinements will be required through future studies. Subsequent studies will benefit from the analysis using a more comprehensive observational pollutant concentrations data set; application of the model over larger urban areas in the region; and application of the coupled street‐scale air quality modeling system to similar urban cities. In addition, a more advanced emission preparation methodology (Lam et al., 2021) could be applied to minimize the uncertainties associated with the emission inventory, and more elaborate emission sources could be modeled explicitly in the ADMS‐Urban model; for example, industrial stacks (Hood et al., 2018). As meteorological factors (e.g., wind) are of great importance to coupled model simulations (Wang, Guo, et al., 2019), improving the representation of urban morphological data in the model could improve baseline model biases. Finally, assessing reduction in the NO_x_/VOC ratio in various areas of a city or in different cities should be cautiously assessed for efficient complex co‐photochemical controls.

## Conflict of Interest

The authors declare no conflicts of interest relevant to this study.

## Supporting information

Supporting Information S1

## Data Availability

The local emission inventory data for running the SMOKE model in the study are available at the Hong Kong EPD website via https://www.epd.gov.hk/epd/english/environmentinhk/air/data/emission_inve.html (Hong Kong Environmental Protection Department, 2019). The observational data for comparing the CMAQ and ADMS‐Urban model simulations are available at the Hong Kong EPD website via https://www.epd.gov.hk/epd/english/environmentinhk/air/data/air_data.html (Hong Kong Environmental Protection Department, 2021) and at the China National Environmental Monitoring Centre website via https://air.cnemc.cn:18,007 with Internet Explorer 10 above and Microsoft Silverlight plug‐in installed (China National Environmental Monitoring Center, 2021). The OpenStreetMap data for providing the road network are available at the OpenStreetMap contributors' website via http://openstreetmap.org/ (Open Street Map Contributors, 2021). The Multi‐resolution Emission Inventory for China (MEIC) for driving the CMAQ model can refer to the released data set from Tsinghua University (Tong et al., 2020).
