# Supplementary material for: Implications of Mitigating Ozone and Fine Particulate Matter Pollution in the Guangdong‐Hong Kong‐Macau Greater Bay Area of China Using a Regional‐To‐Local Coupling Model
Source: Geohealth. 2022 Mar 11;6(3):e2021GH000506. doi: 10.1029/2021GH000506 (PMC8914409; doi:10.1029/2021GH000506)
Supplement: Supplementary file 1 — Supporting Information S1 [file GH2-6-e2021GH000506-s001.pdf]

*GeoHealth Journal*

Supporting Information for

# Implications of Mitigating Ozone and Fine Particulate Matter Pollution in the Guangdong-Hong Kong-Macau Greater Bay Area of China Using a Regional-to-Local Coupling Model

Xuguo Zhang[1,2], Jenny Stocker[3], Kate Jonhnson[3], Yik Him Fung[2], Teng Yao[2], Christina Hood[3], David Carruthers[3], and Jimmy C. H. Fung[1,2,*]

[1]Department of Mathematics, The Hong Kong University of Science and Technology, Hong Kong

[2]Division of Environment and Sustainability, The Hong Kong University of Science and Technology, Hong Kong

[3]Cambridge Environmental Research Consultants, Cambridge, UK.

Corresponding author: Jimmy C. H. Fung (majfung@ust.hk)

ORCID: 0000-0001-7857-2525 (X. Zhang); 0000-0003-3243-7226 (J. Stocker); 0000-0002-7071-1137 (Y.H. Fung); 0000-0001-9244-5696 (C. Hood); 0000-0002-7859-8511 (J.C.H. Fung)

## Contents of this file

Figures S1 to S15.

## Introduction

The supporting information consists of 18 pages, including 15 figures. Besides the research framework and the model domain setting are shown in Figures S1, and S2, some example emission plots for the 1 km domain are shown in Figures S3–S8. All emissions presented are given as “daily column” values, i.e., the values correspond to daily average emissions summed over all vertical levels included in the modeled 3D emissions grids. In the regional model, VOCs are a complex mixture of different components. For the emissions plots shown here, paraffin (PAR) emissions have been used as a species representative of total anthropogenic VOCs. Figure S4 compares the total PAR emissions for the BAU case and the three scenarios. Visually, the reduction in PAR emissions on the roads can be seen by comparing Figures S4a and S4b, i.e., the signature of the road sources is reduced; comparing Figures S4a and S4c highlights the reduction in industrial source emissions; and Figure S4d shows the result of both reductions. Figure S5 presents spatial plots of differences that emphasize the changes in emissions. Figure S5a clearly indicates reductions near-road networks in the difference between the base case and the reduced traffic scenario. At the same time, Figure S5b shows large reductions at locations where there is intensive industrial activity (Shenzhen, Dongguan, and Guangzhou). Similar patterns of $NO_x$ and $PM_{2.5}$ can be assessed in Figures S6 and S8, respectively.

**List of Supplemental Figures**

**Figure S1.** Research framework for the CMAQ–ADMS-Urban modeling system.

**Figure S2.** Geographic domain setting of the CMAQ–ADMS-Urban modeling system: meso-scale meteorological model WRF (blue lines) and regional chemical transport model CMAQ (red lines) for D1 (27 km), D2 (9 km), D3 (3 km), D4 (1 km) and street-level ADMS-Urban model (purple lines) for D5. The Greater Bay Area (GBA) includes the PRD Economic Zone (Guangzhou (GZ), Shenzhen (SZ), Foshan (FS), Dongguan (DG), Zhuhai (ZH), Zhongshan (ZS), Jiangmen (JM), Huizhou (HZ), Zhaoqing (ZQ)), Hong Kong (HK), and Macau (MC).

**Figure S3.** Emissions for the inner regional model domain covering central GBA for (a) $NO_x$, (b) VOC, and (c) $PM_{2.5}$. Unit: thousand tonnes per year.

**Figure S4.** Daily column emission comparison of anthropogenic PAR (model species representative of total VOC) for (a) Base case, (b) Half Traffic case, (c) Half Industry VOC case, (d) Both control case. Unit: moles/s.

**Figure S5.** Daily column emission difference plots for anthropogenic PAR for (a) Half Traffic minus Base case, (b) Half Industry VOC minus Base case, and (c) Both controls minus Base case. Unit: moles/s.

**Figure S6.** Daily column emission comparison of anthropogenic NOx for (a) Base case, (b) Half Traffic case, (c) Half Industry VOC case, (4) Both control case. Unit: moles/s.

**Figure S7.** Daily column emission difference plots for anthropogenic NOx for (a) Half Traffic minus Base case (b) Half Industry VOC minus Base case, (c) Both controls minus Base case. Unit: moles/s.

**Figure S8.** Daily column emission comparison of anthropogenic $PM_{2.5}$ for (a) Base case, (b) Half Traffic case, (c) Half Industry VOC case, and (d) Both control case. Unit: g/s.

**Figure S9.** Daily column emission difference plots of anthropogenic $PM_{2.5}$ for (a) Half Traffic minus Base case (b) Half Industry VOC minus Base case, (c) Both controls minus Base case. Unit: g/s.

**Figure S10.** Time series comparison of $NO_2$ for typical stations in the GBA from the CMAQ model outputs. Unit: ppb.

**Figure S11.** Time series comparison of $O_3$ for typical stations in the GBA from the CMAQ model output. Unit: ppb.

**Figure S12**. Time series comparison of $PM_{2.5}$ for typical stations in the GBA from the CMAQ model output. Unit: $\mu g/m^3$.

**Figure S13.** Time series comparison of $NO_2$ in urban and roadside stations for the CMAQ base case (blue line) and the ADMS-Urban base case (green line). Unit: $\mu g/m^3$.

**Figure S14.** Time series comparison of $O_3$ in urban and roadside stations for the CMAQ base case (blue line) and the ADMS-Urban base case (green line). Unit: $\mu g/m^3$.

**Figure S15.** Time series comparison of $PM_{2.5}$ in urban and roadside stations for the CMAQ base case (blue line) and the ADMS-Urban base case (green line). Unit: $\mu g/m^3$.

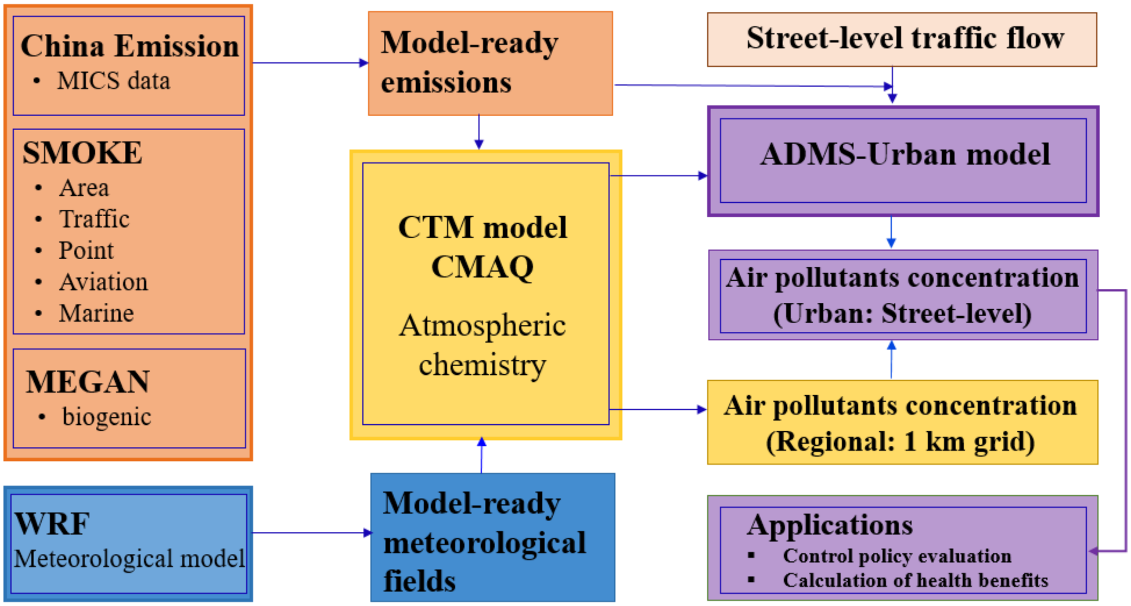


**Figure S1.** Research framework for the CMAQ–ADMS-Urban modeling system.

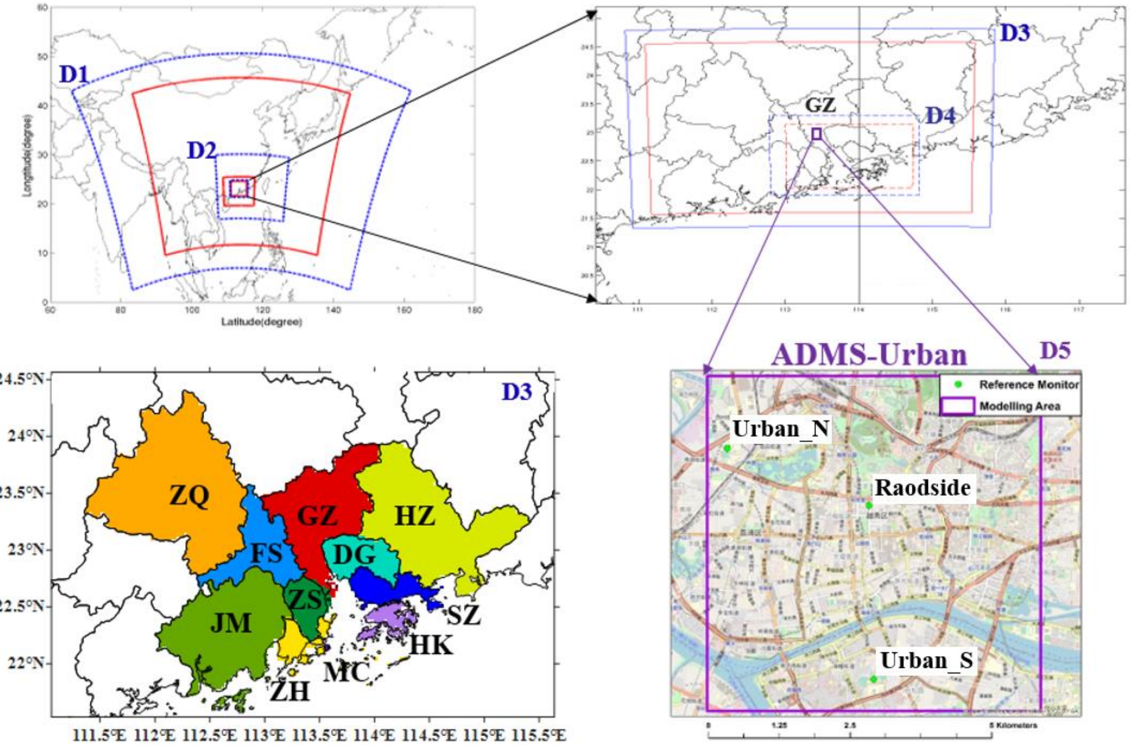


**Figure S2.** Geographic domain setting of the CMAQ–ADMS-Urban modeling system: meso-scale meteorological model WRF (blue lines) and regional chemical transport model CMAQ (red lines) for D1 (27 km), D2 (9 km), D3 (3 km), D4 (1 km) and street-level ADMS-Urban model (purple lines) for D5 (6 km × 6 km). The Greater Bay Area (GBA) includes the PRD Economic Zone (Guangzhou (GZ), Shenzhen (SZ), Foshan (FS), Dongguan (DG), Zhuhai (ZH), Zhongshan (ZS), Jiangmen (JM), Huizhou (HZ), Zhaoqing (ZQ)), Hong Kong (HK), and Macau (MC).

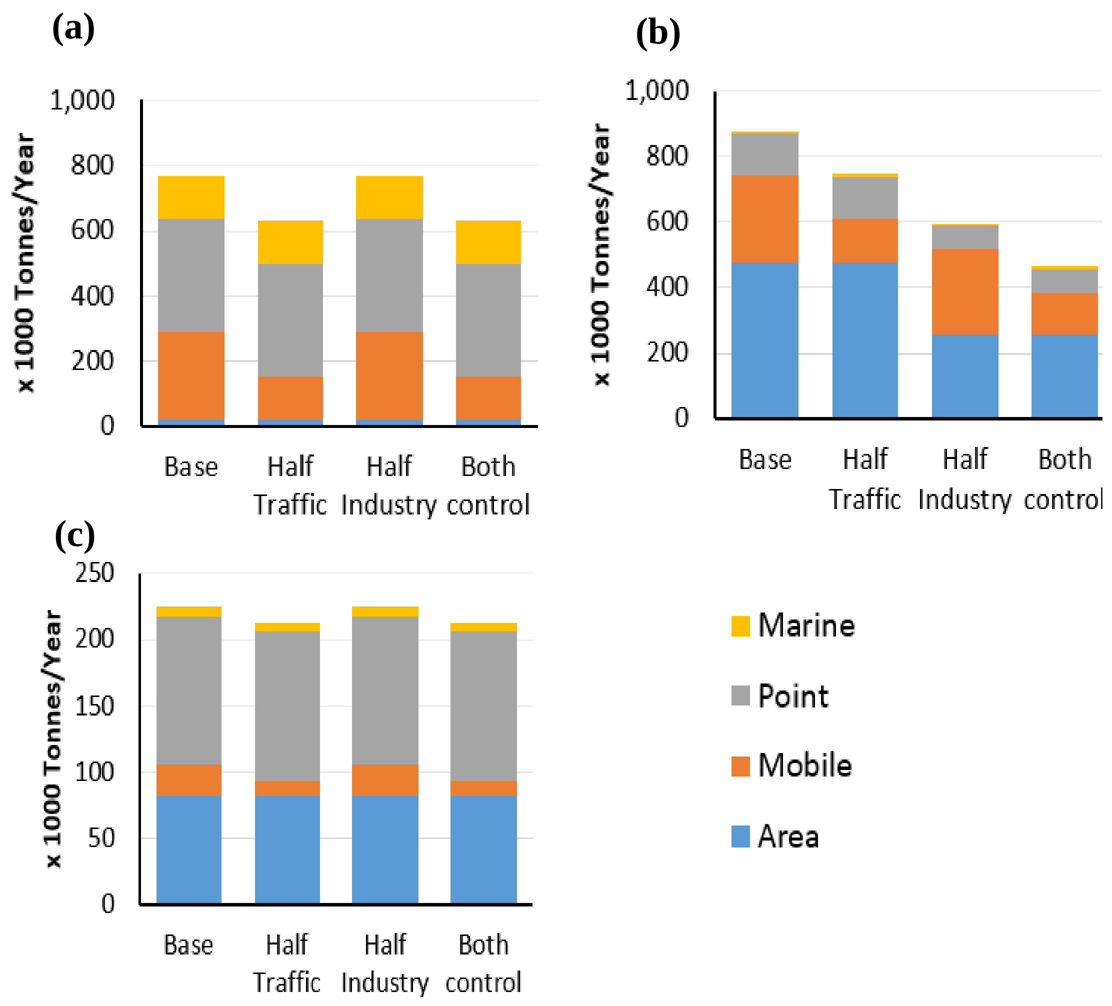


**Figure S3.** Emissions for the inner regional model domain covering central GBA for (a) $NO_x$, (b) VOC, and (c) $PM_{2.5}$. Unit: thousand tonnes per year.

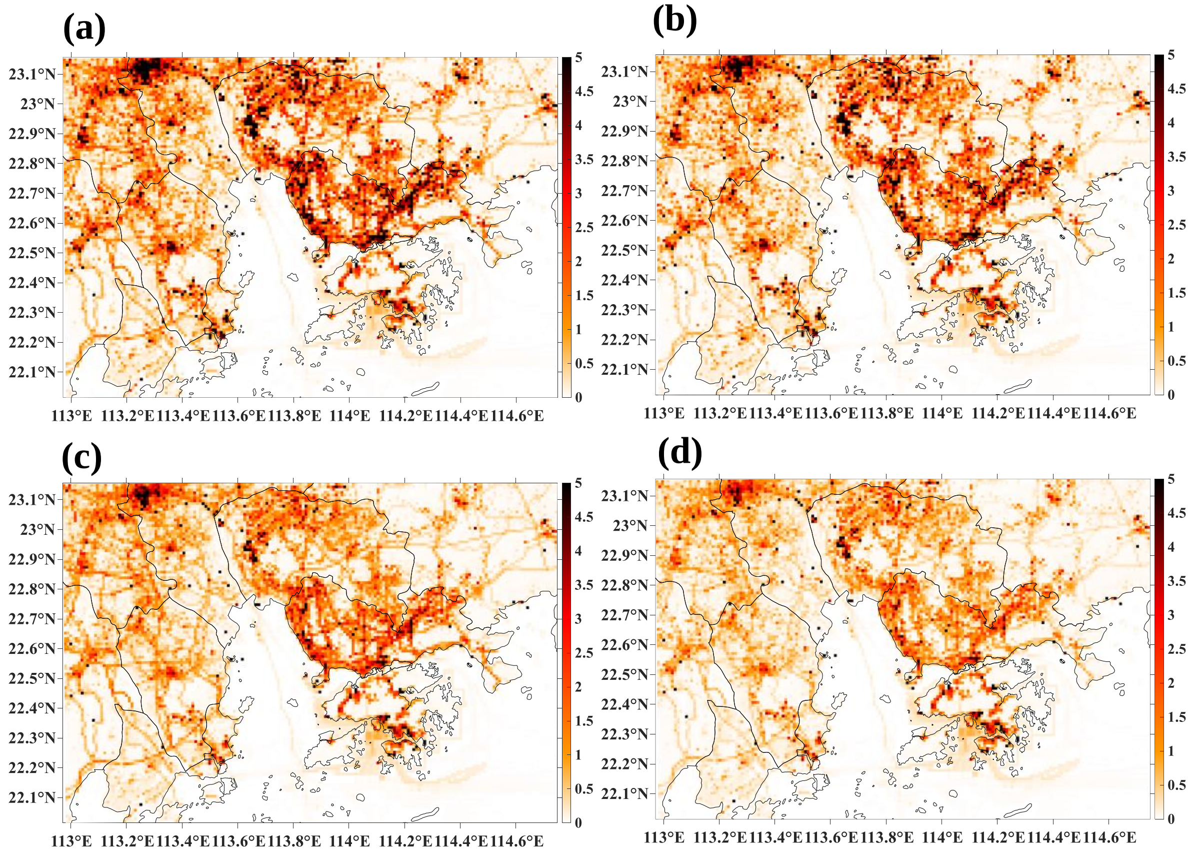


**Figure S4.** Daily column emission comparison of anthropogenic PAR (model species representative of total VOC) for (a) Base case, (b) Half Traffic case, (c) Half Industry VOC case, (d) Both control case. Unit: moles/s.

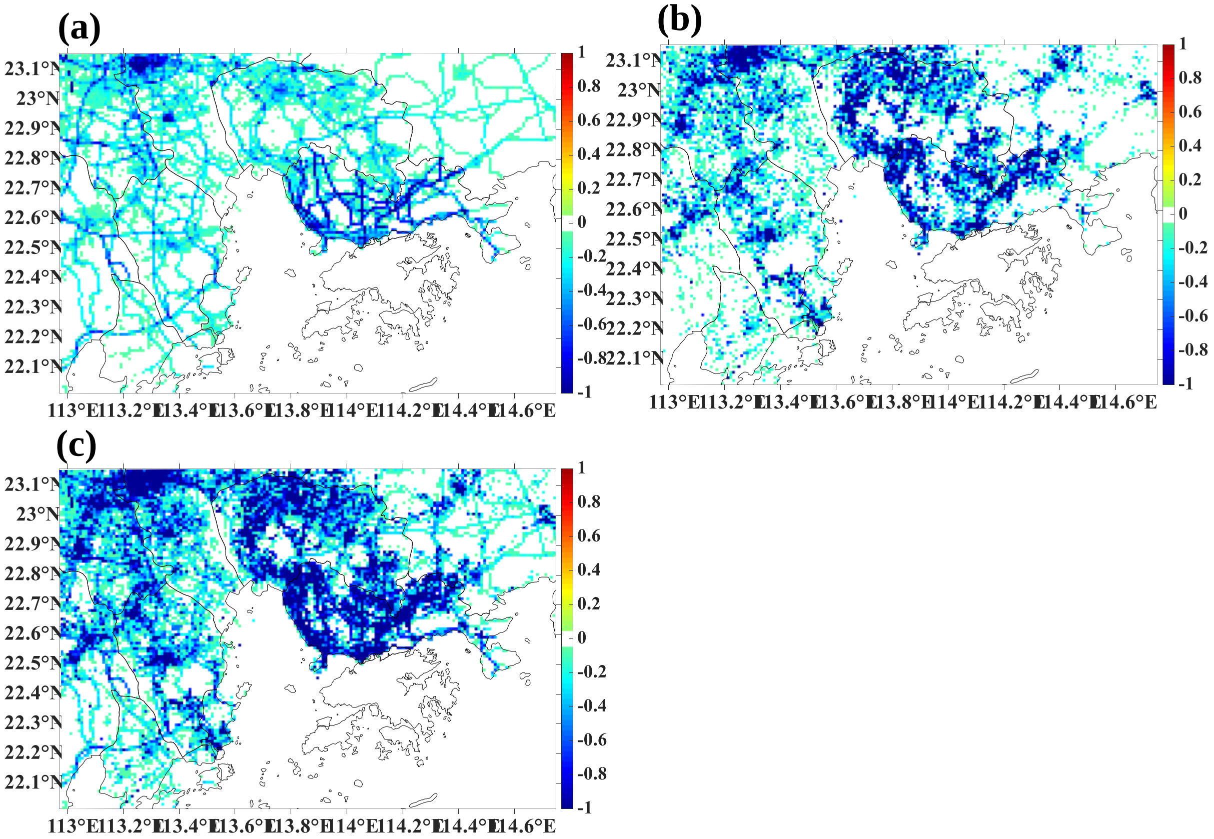


**Figure S5.** Daily column emission difference plots for anthropogenic PAR for (a) Half Traffic minus Base case, (b) Half Industry VOC minus Base case, and (c) Both controls minus Base case. Unit: moles/s.

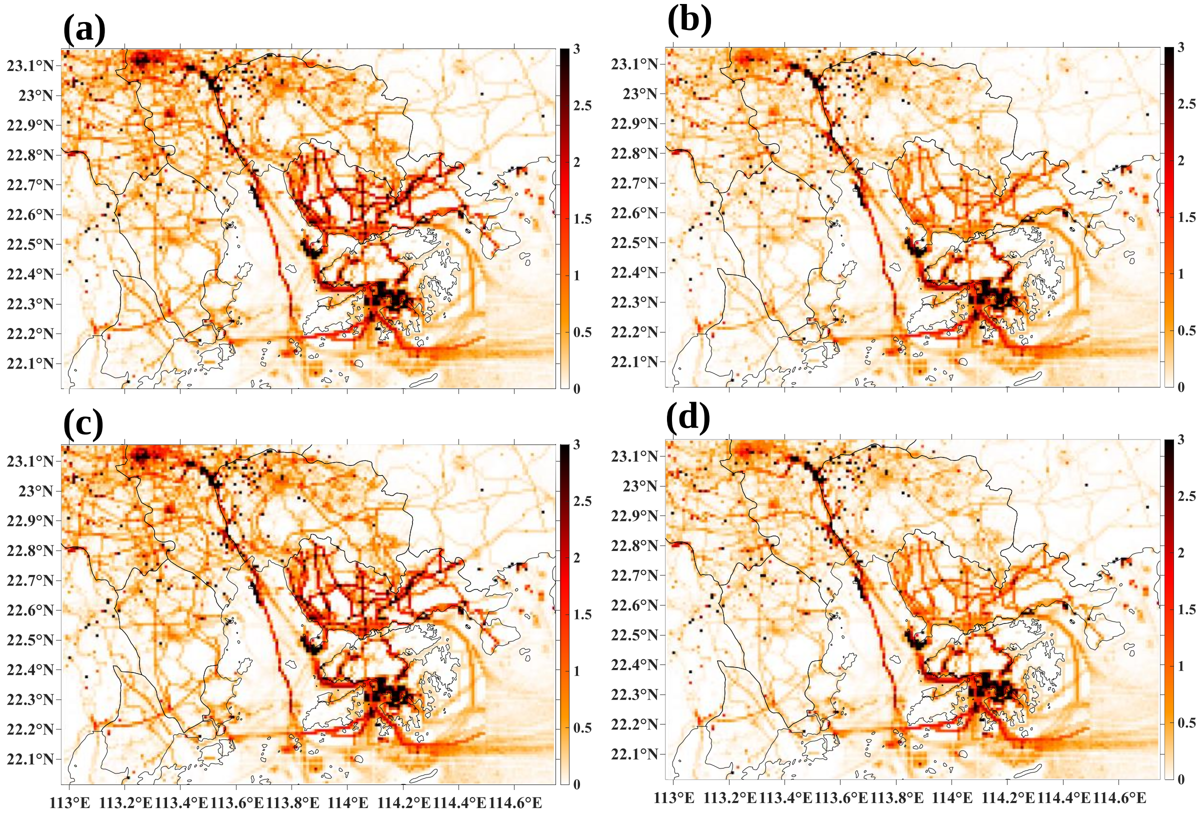


**Figure S6.** Daily column emission comparison of anthropogenic NOx for (a) Base case, (b) Half Traffic case, (c) Half Industry VOC case, (4) Both control case. Unit: moles/s.

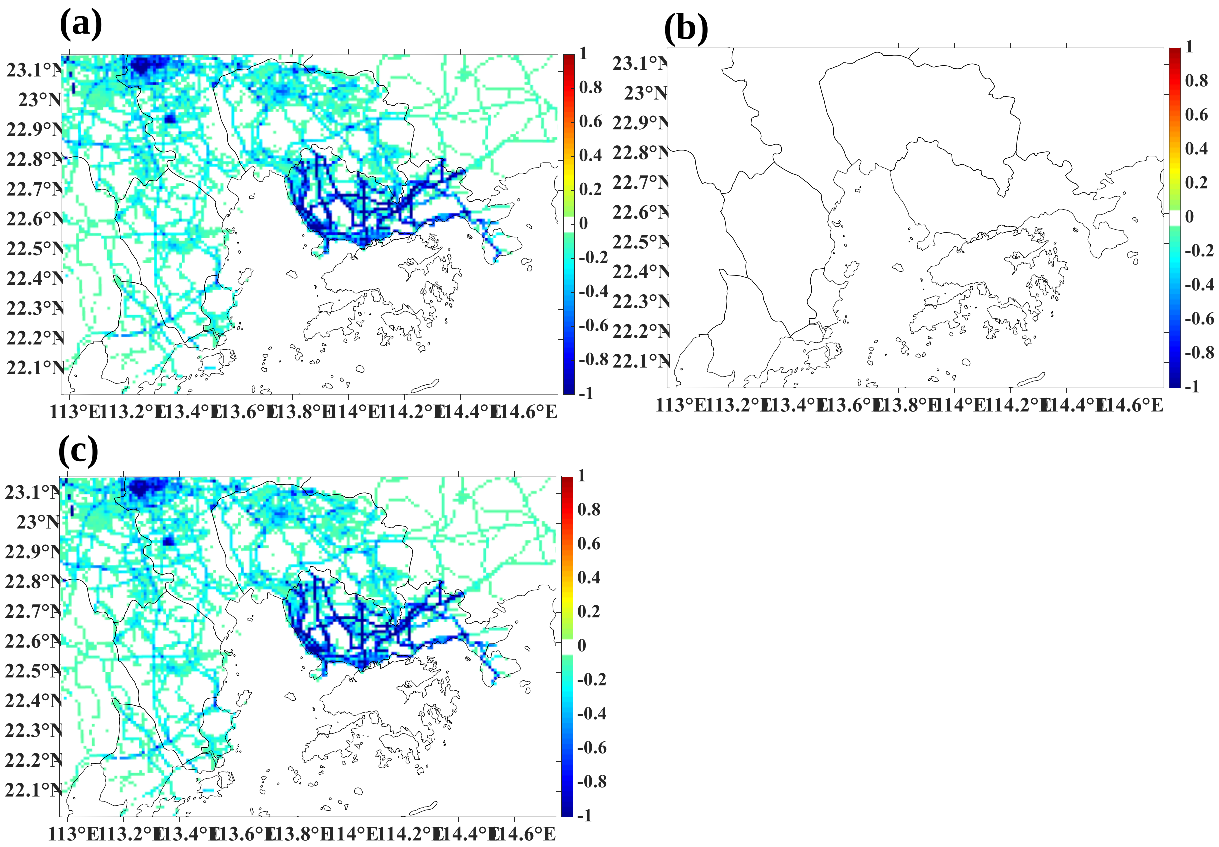


**Figure S7.** Daily column emission difference plots for anthropogenic NOx for (a) Half Traffic minus Base case (b) Half Industry VOC minus Base case, (c) Both controls minus Base case. Unit: moles/s.

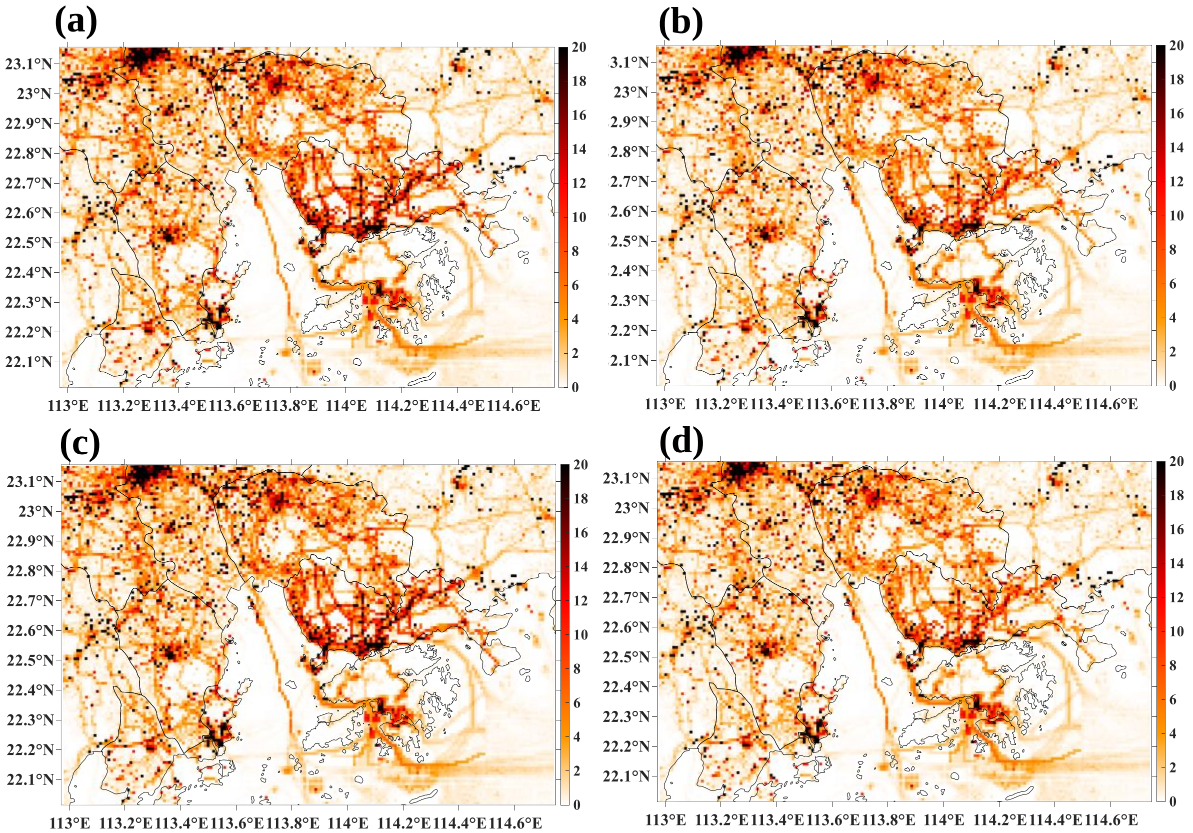


**Figure S8.** Daily column emission comparison of anthropogenic $PM_{2.5}$ for (a) Base case, (b) Half Traffic case, (c) Half Industry VOC case, and (d) Both control case. Unit: g/s.

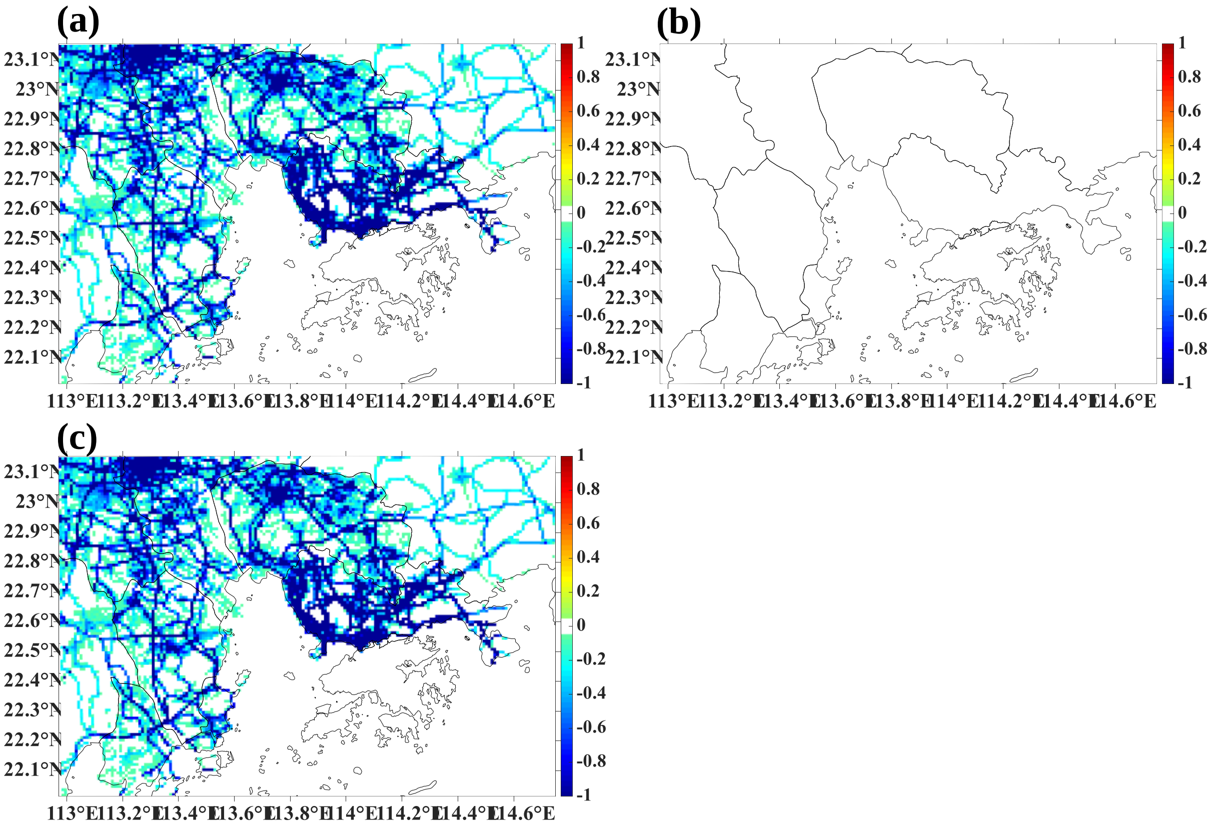


**Figure S9.** Daily column emission difference plots of anthropogenic $PM_{2.5}$ for (a) Half Traffic minus Base case (b) Half Industry VOC minus Base case, (c) Both controls minus Base case. Unit: g/s.

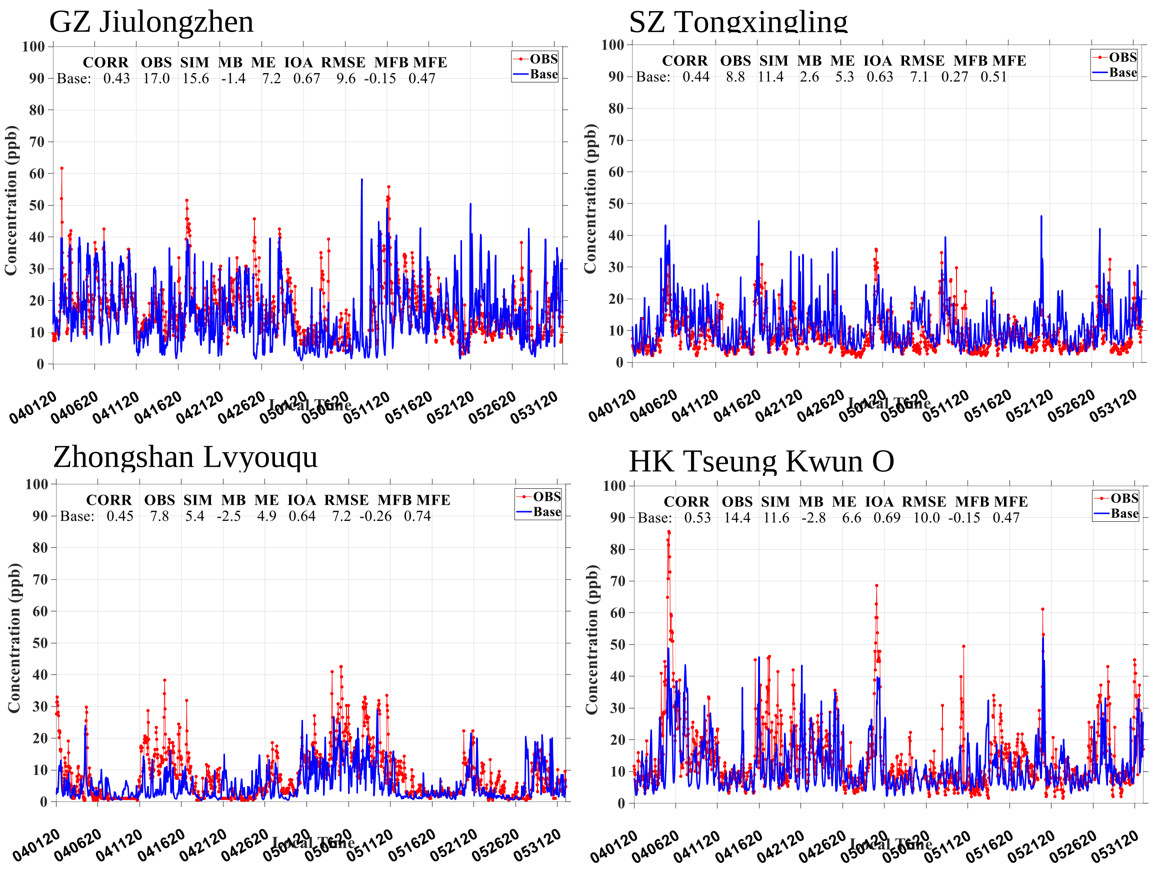


**Figure S10.** Time series comparison of $NO_2$ for typical stations in the GBA from the CMAQ model outputs. Unit: ppb.

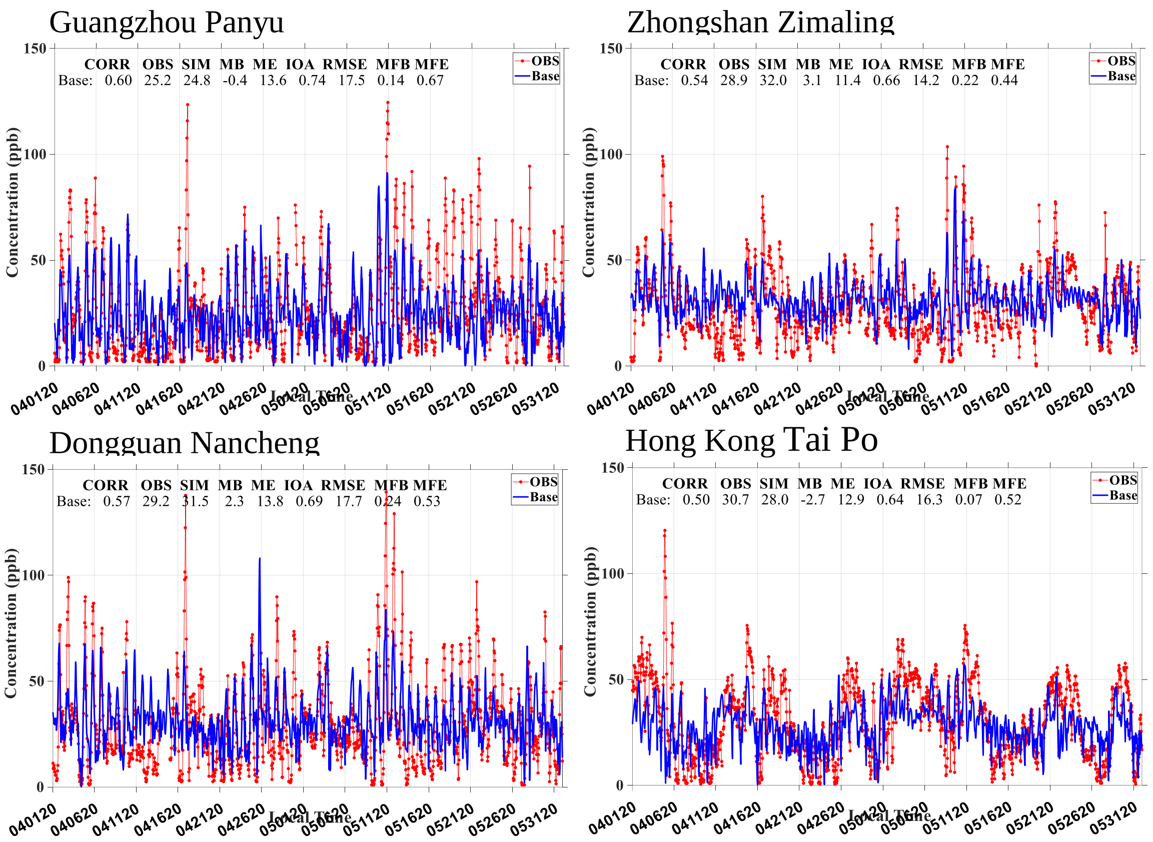


**Figure S11.** Time series comparison of $O_3$ for typical stations in the GBA from the CMAQ model output. Unit: ppb.

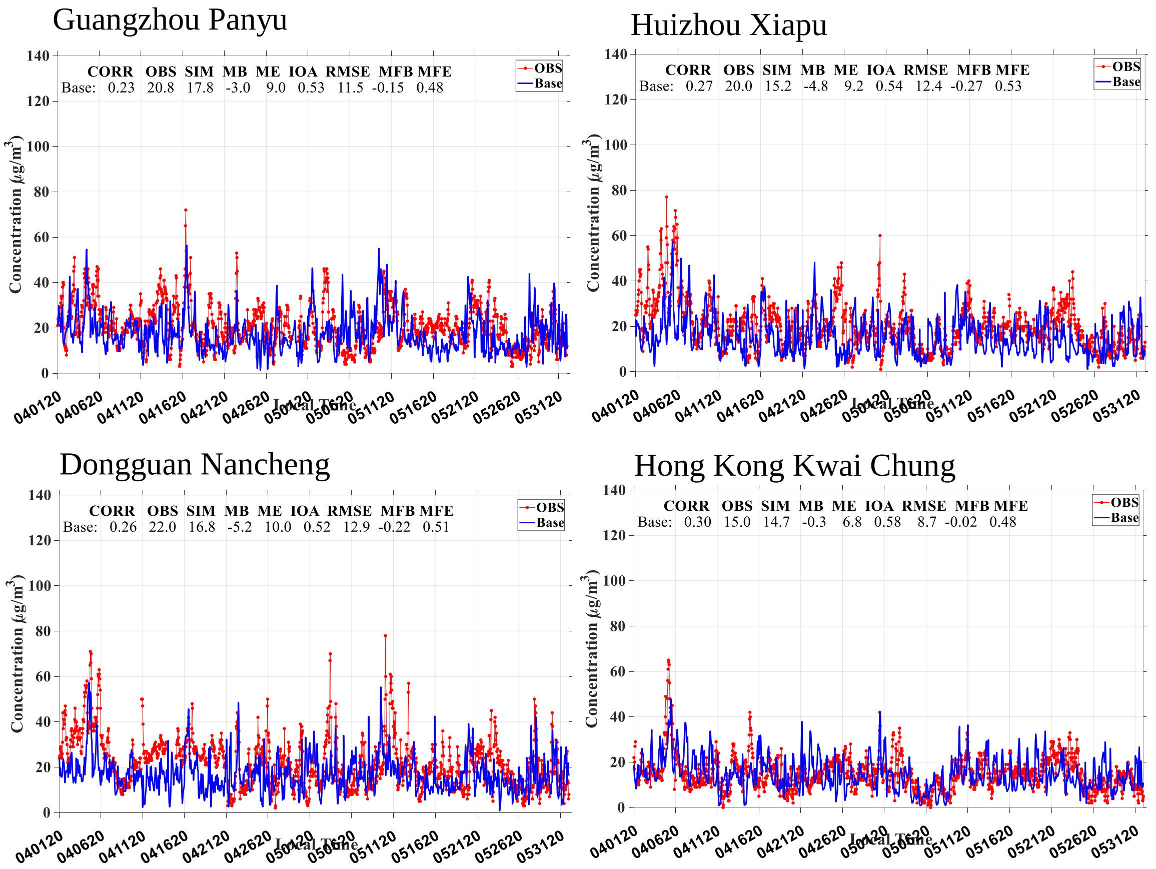


**Figure S12**. Time series comparison of $PM_{2.5}$ for typical stations in the GBA from the CMAQ model output. Unit: μg/m$^3$.

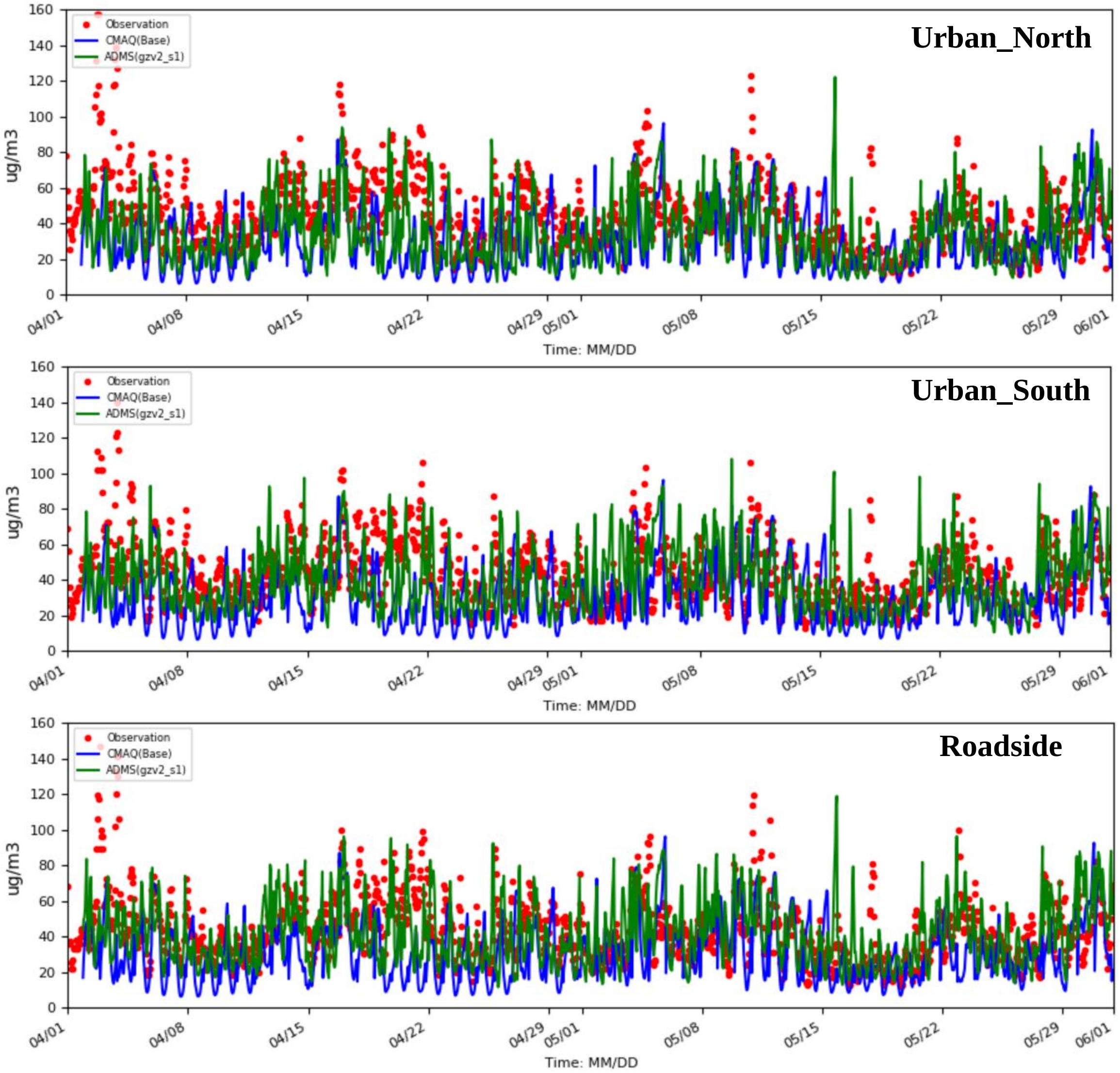


**Figure S13**. Time series comparison of $NO_2$ in urban and roadside stations for the CMAQ base case (blue line) and the ADMS-Urban base case (green line). Unit: $\mu g/m^3$.

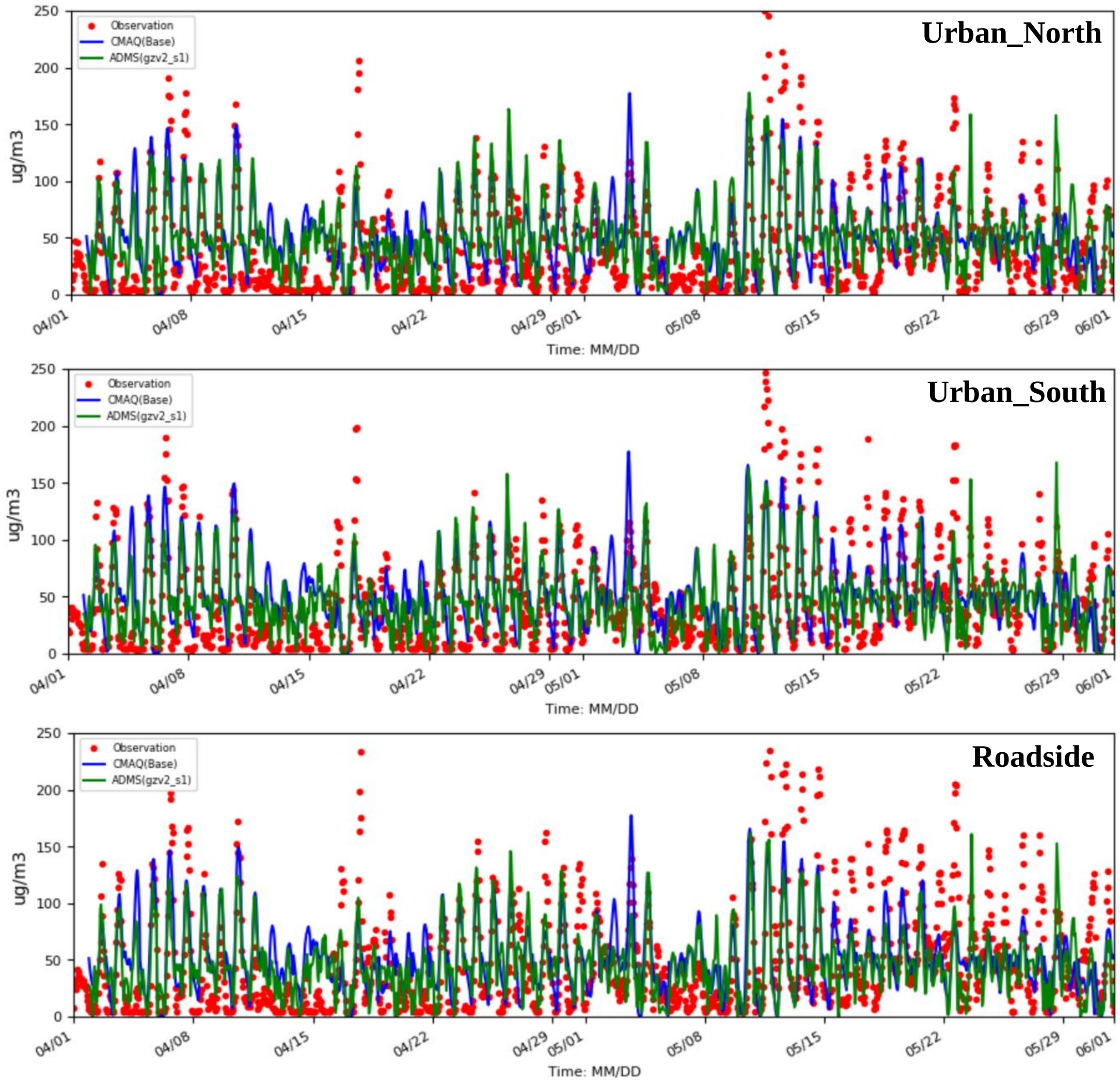


**Figure S14.** Time series comparison of $O_3$ in urban and roadside stations for the CMAQ base case (blue line) and the ADMS-Urban base case (green line). Unit: $\mu g/m^3$.

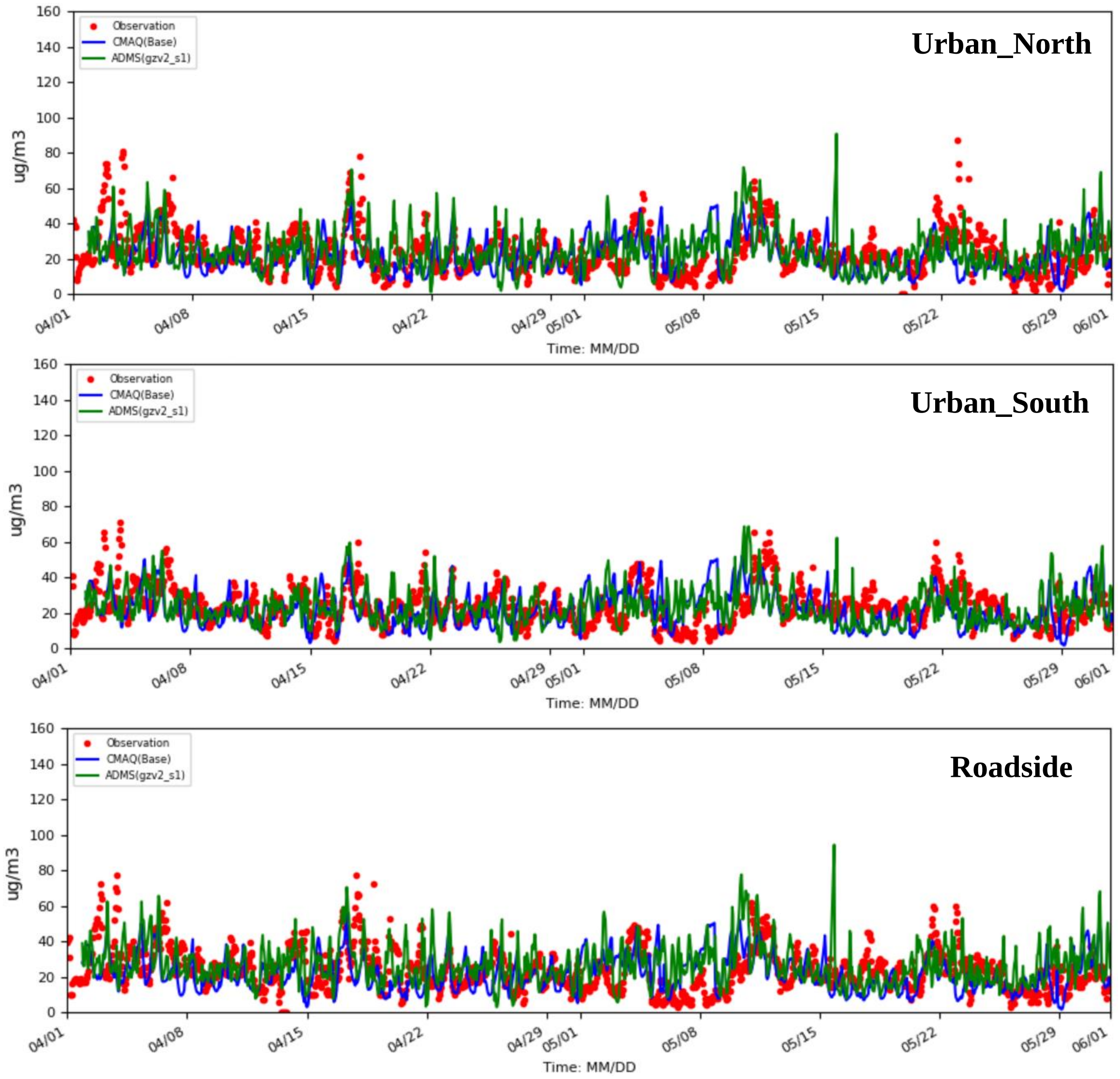


**Figure S15.** Time series comparison of $PM_{2.5}$ in urban and roadside stations for the CMAQ base case (blue line) and the ADMS-Urban base case (green line). Unit: μg/m$^3$.
